# Data in support of global role of the membrane protease LonB in *Archaea*: Potential protease targets revealed by quantitative proteome analysis of a *lonB* mutant in *Haloferax volcanii*

**DOI:** 10.1016/j.dib.2015.04.013

**Published:** 2015-05-07

**Authors:** Micaela Cerletti, Roberto A. Paggi, Carina Ramallo Guevara, Ansgar Poetsch, Rosana E. De Castro

**Affiliations:** a Instituto de Investigaciones Biológicas, Universidad Nacional de Mar del Plata (UNMDP), Consejo Nacional de Investigaciones Científicas y Técnicas (CONICET), Funes 3250 4to nivel, Mar del Plata 7600, Argentina; b Plant Biochemistry, Ruhr University Bochum, 44801 Bochum, Germany

## Abstract

This data article provides information in support of the research article “Global role of the membrane protease LonB in *Archaea*: Potential protease targets revealed by quantitative proteome analysis of a *lonB* mutant in *Haloferax volcanii*” [1]. The proteome composition of a wt and a LonB protease mutant strain (suboptimal expression) in the archaeon *Haloferax volcanii* was assessed by a quantitative shotgun proteomic approach. Membrane and cytosol fractions of *H. volcanii* strains were examined at two different growth stages (exponential and stationary phase). Data is supplied in the present article. This study represents the first proteome examination of a Lon-deficient cell of the *Archaea* Domain.

Specifications table

**Table:** 

Subject area	*Biology*
More specific subject area	Microbiology, Archaeal Physiology
Type of data	Proteome Discoverer search results (xls), Tables (pdf), *H. volcanii* proteome database (FASTA)
How data was acquired	One-dimensional nLC–ESI-MS/MS using instruments LTQ Orbitrap XL for cytoplasm samples and LTQ Orbitrap Elite for membrane samples
Data format	Processed
Experimental factors	*H. volcanii* wt and a LonB protease conditional mutant strain (down regulation) (HVLON3) were used for proteome analysis. Cultures were examined in the exponential and stationary growth phases
Experimental features	Cytoplasm and membrane proteins electrophoresed in 1D gels, all proteins were concentrated into one protein band; protein bands excised and digested with trypsin. Tryptic peptides eluted and subjected to nLC-ESI-MS/MS
Data source location	N/A
Data accessibility	Data is provided in Supplementary material directly with this article

Value of the data

– First proteome determination of an archaeon deficient in the membrane-bound LonB protease.
– Unique proteins were detected in wt and *lon* mutant strains at different growth phases.
– Comprehensive information on the 1778 proteins detected in each strain and growth condition.
– Insights is provided on the relevance of the Lon protease in archaeal physiology.

## 1 Data, experimental design, materials and methods

Applying a quantitative shotgun proteomics approach (One-dimensional nLC-ESI-MS/MS) we have obtained insight on the proteome composition of the haloarchaeon *H. volcanii* H26 wt vs a conditional mutant that synthesizes suboptimal amounts of the membrane protease LonB (HVLON3). Supplementary Table S1 shows a list of all the proteins that were identified combining these strains, organized according to their functional category. A total of 1778 proteins were detected (including membrane and cytoplasm fractions) representing 44% of the predicted *H. volcanii* theoretical proteome. Additionally, this data allowed the identification of the unique proteins detected in each growth phase and/or strain ( Supplementary Table S2). Supplementary Table S3 (1–4) shows the Proteome Discoverer database search results for all the replicates (4) of the wt, HVLON3 and HVABI strains.

### 1.1 Strains and culture conditions

*H. volcanii* H26 wt and the mutant strain HVLON3 were used for proteome comparison. HVLON3 is a conditional expression strain which has the tryptophan-regulated promoter (P*tnaA*) [2] located upstream the *lon* gene in *H. volcanii* H26 chromosome (P*tnaA*-*lon-abi)*. This strain synthesizes very low amounts of Lon and Abi in absence of trp in the culture medium [3]. As a control, the proteome of the strain HVABI, a deletion mutant of the downstream gene *abi* (Δ*abi*) [3] was analyzed in parallel and compared to that of the wt strain. To obtain the “Lon subproteome”, proteins that changed as a consequence of the Abi mutation were discarded. These strains were grown in minimal medium (Hv-Min) containing uracil (50 μg ml^−1^) [4] in absence of trp at 42 °C 200 rpm. Cell growth was monitored by measuring the optical density of the cultures at 600 nm (OD_600_). Samples were taken at exponential (Exp) (OD_600_~0.5) and stationary (St) (OD_600_~1.5) growth phases. For proteome analysis four independent cultures (biological replicates) of each strain were analyzed and compared.

### 1.2 Preparation of cytoplasm and membrane fractions

Cells were harvested by centrifugation (10,000×*g*, 10 min, 4 °C), suspended in 100 mM HCl–Tris (pH 7.5) containing 2 M NaCl and disrupted with an ultrasonic processor (3×30 s, 80 W). Cell lysates were clarified by centrifugation (10,000×*g*, 10 min, 4 °C) and the membranes pelleted (100,000×*g*, 2 h, 4 °C) and washed with the same buffer (100,000×g, 30 min, 4 °C). The membranes were suspended in 1/3 of the same buffer (~ 1 ml). To eliminate salts, cytoplasm and membrane proteins were precipitated overnight with 100% (v/v) acetone at 4 °C followed by centrifugation. The precipitated proteins were washed three times with 80% and once with 100% acetone and left to dry for a few minutes at room temperature.

### 1.3 Electrophoresis in polyacrylamide gels (SDS-PAGE)

Cytoplasm and membrane proteins were suspended in 1X Laemmli sample buffer (12 mM Tris–HCl pH 6.8, 0.4% (w/v) SDS, 0.02% (w/v) bromophenol blue (BPB), 0.1 M DTT, 5% (v/v) glycerol), incubated at 37 °C for 3 h (550 rpm) and loaded onto 10% (v/v) polyacrylamide gels containing 0.1% SDS (~ 30 μg per lane). The gels were run at room temperature until samples passed the stacking gel and all proteins were concentrated into one protein band in the separation gel. Proteins were visualized with a coomassie brilliant blue (CBB-G250) stain as described by Dyballa and Metzger [5].

### 1.4 In-gel tryptic digestion

Protein bands were excised from the gels and cut into small cubes (ca. 1×1 mm) which were completely destained according to Schlüesener and colleagues [6]. Gel pieces were dried in a SpeedVac, trypsin (porcine, sequencing grade, Promega) solution (12.5 ng ml^−1^ in 25 mM ammonium bicarbonate, pH 8.6) was added until gel pieces were immersed completely in digestion solution. The protein digestion was performed ON at 37 °C with agitation (tempered shaker HLC MHR20, 550 rpm). After digestion, elution buffer (50% acetonitrile, 0.5% TFA, UPLC grade, Biosolve, Netherlands) was added (1 µl elution buffer for each µl of digestion buffer) and the samples were sonicated for 20 min in an ultrasonic bath. The samples were centrifuged and the supernatants were transferred to new 1.5 ml tubes. The extracted peptides were dried using a SpeedVac and stored at −20 °C. Before MS-analysis peptides were re-suspended in 20 µl of buffer A (0.1% formic acid in water, ULC/MS, Biosolve, Netherlands) by sonication for 10 min and transferred to LC–MS grade glass vials (12×32 mm^2^ glass screw neck vial, Waters, USA). Each measurement was performed with 8 μl of sample.

### 1.5 One-dimensional nLC-ESI-MS/MS

An UPLC HSS T3 column (1.8 µm, 75 µm×150 mm, Waters, Milford, MA, USA) and an UPLC Symmetry C_18_ trapping column (5 µm, 180 µm×20 mm, Waters, Milford, MA, USA) for LC as well as a PicoTip Emitter (SilicaTip, 10 µm i.d., New Objective, Woburn, MA, USA) were used in combination with the nanoACQUITY gradient UPLC pump system (Waters, Milford, MA, USA) coupled to a LTQ Orbitrap XL (analyzing the cytoplasm samples) or a LTQ Orbitrap Elite (analyzing the membrane samples) mass spectrometer (Thermo Fisher Scientific Inc., Waltham, MA, USA). For elution of the peptides a linear gradient with increasing concentration of buffer B (0.1% formic acid in acetonitrile, ULC/MS, Biosolve, Netherlands) from 1% to 40% within 165 min was applied, followed by a linear gradient from 40% to 99% acetonitrile concentration within 15 min (0–5 min: 1% buffer B; 5–10 min: 5% buffer B; 10–165 min: 40% buffer B; 165–180 min: 99% buffer B; 180–195 min: 1% buffer B) at a flow rate of 400 nL min^−1^ and a spray voltage of 1.5–1.8 kV. The column was re-equilibrated at 1% buffer B within 15 min. The analytical column oven was set to 55 °C and the heated desolvation capillary was set to 200 °C (XL) or 275 °C (Elite). The LTQ XL Orbitrap was operated by instrument method files of Xcalibur (Rev. 2.0.7) and the LTQ Orbitrap Elite via instrument method files of Xcalibur (Rev. 2.1.0) in positive ion mode. The linear ion trap and Orbitrap were operated in parallel, i.e. during a full MS scan on the Orbitrap in the range of 300–1600 *m/z* (XL) or 150–2000 *m/z* (Elite) at a resolution of 60,000 MS/MS spectra of the 10 most intense precursors, from most intense to least intense, were detected in the ion trap. All samples were re-analyzed, but with reverse order of the 10 most intense precursor fragmentations, i.e. from least intense to most intense. All the measurements in the Orbitrap Elite were performed with the lock mass option (lock mass: *m/z* 445.120025) for internal calibration [7]. The relative collision energy for collision-induced dissociation (CID) was set to 35%. Dynamic exclusion was enabled with a repeat count of 1 and 60 s (XL) or 45 s exclusion duration window (Elite). Singly charged and ions of unknown charge state were rejected from MS/MS.

### 1.6 Protein identification

Protein identification was performed by SEQUEST [8] and MS Amanda [9] algorithms embedded in Proteome Discoverer 1.4 (Thermo Electron^©^ 2008-2012) searching against the complete proteome database of *H. volcanii DS2* containing 4035 entries exported from the Halolex database [10] on 9/24/2013 (Hfvol_prot file). The mass tolerance for precursor ions was set to 15 ppm (XL) or 7 ppm (Elite); the mass tolerance for fragment ions was set to 0.4 Da. Only tryptic peptides with up to two missed cleavages were accepted and the oxidation of methionine was admitted as a variable peptide modification. The false discovery rate (FDR) was determined with the percolator validation in Proteome Discoverer 1.4 and the *q*-value was set to 1% [11]. For protein identification the mass spec format-(msf)-files were filtered with peptide confidence “high” and two unique peptides per protein. Additionally protein grouping options were enabled as default, which means consider only PSMs with confidence at least “medium” and consider only PSMs with delta CN better than 0.15.

## Supplementary materials

Supplementary material: Supplementary Table S3**(1–4): Proteome Discoverer database search of all the replicates of wt, HVLON3 and HVABI strains.** The data in the different files is distributed as follow: **S3-1.** Cytoplasmic fraction exponential phase. **S3-2.** Cytoplasmic fraction stationary phase. **S3-3.** Membrane fraction exponential phase. **S3-4.** Membrane fraction stationary phase. The replicates for each condition are named A to D in the file sheet. **Accession:** unique identifier assigned to the protein by the *H. volcanii* FASTA database. **Description:** name of the protein. **∑Coverage:** percentage of the protein sequence covered by identified peptides. **∑# Proteins:** number of identified proteins in the protein group of a master protein. **∑# Unique Peptides:** number of peptide sequences unique to a protein group. **∑# Peptides:** total number of peptide matches found during the search. **∑# PSMs:** total number of identified peptide sequences (peptide spectrum matches) for the protein, including those redundantly identified. **Area:** average area of the three unique peptides with the largest peak area. **Score:** protein score, which is the sum of the scores of the individual peptides. **A2:** Results from Sequest HT. **A4:** Results from MSAmanda. **# AAs:** sequence length of the protein. **MW [Da]:** calculated molecular weight of the protein. **calc. pI:** theoretically calculated isoelectric point.
