# Supplementary material for: Data in support of global role of the membrane protease LonB in Archaea: Potential protease targets revealed by quantitative proteome analysis of a lonB mutant in Haloferax volcanii
Source: Data Brief. 2015 May 7;4:50–3. doi: 10.1016/j.dib.2015.04.013 (PMC4510384; doi:10.1016/j.dib.2015.04.013)
Supplement: Supplementary file 1 — Supplementary material: Supplementary Table S3(1–4): Proteome Discoverer database search of all the replicates of wt, HVLON3 and HVABI strains. The data in the different files is distributed as follow: S3-1. Cytoplasmic fraction exponential phase. S3-2. Cytoplasmic fraction stationary phase. S3-3. Membrane fraction exponential phase. S3-4. Membrane fraction stationary phase. The replicates for each condition are named A to D in the file sheet. Accession: unique identifier assigned to the protein by the H. volcanii FASTA database. Description: name of the protein. ∑Coverage: percentage of the protein sequence covered by identified peptides. ∑# Proteins: number of identified proteins in the protein group of a master protein. ∑# Unique Peptides: number of peptide sequences unique to a protein group. ∑# Peptides: total number of peptide matches found during the search. ∑# PSMs: total number of identified peptide sequences (peptide spectrum matches) for the protein, including those redundantly identified. Area: average area of the three unique peptides with the largest peak area. Score: protein score, which is the sum of the scores of the individual peptides. A2: Results from Sequest HT. A4: Results from MSAmanda. # AAs: sequence length of the protein. MW [Da]: calculated molecular weight of the protein. calc. pI: theoretically calculated isoelectric point. [file mmc1.zip › Table S1.pdf]

Table S1. List of proteins identified in *H. volcanii* wt and HVLON3 strains. Protein detection was based on a minimum of two unique peptides (in at least one biological replicate) with a minimal false discovery rate of q-value ≤ 1%. FC, functional category (REG: gene regulation, SIG: signal transduction, CHP: chaperones, RMT: RNA maturation, RRR: replication, repair, recombination, TC: transcription, TL: translation, AA: amino acid metabolism, CHM: carbohydrate metabolism, CIM: central intermediary metabolism, COM: coenzyme metabolism, EM: energy metabolism, LIP: lipid metabolism, NUM: nucleotide metabolism, GEN: general enzymatic function, ISH: transposases and ISH-encoded proteins, MIS: miscellaneous, CE: cell envelope, CP: cellular processes, SEC: protein secretion, TP: small molecule transport, CHY: conserved hypothetical protein, HY: hypothetical protein).

ID	Gene name	FC	Protein name
Environmental information processing			
HVO_0083		REG	GlnK-type ammonia transport regulator
HVO_0179	*trh1*	REG	Lrp/AsnC family transcription regulator / TrkA domain protein
HVO_0240	*trh2*	REG	Lrp/AsnC family transcription regulator
HVO_0246		REG	ArsR family transcription regulator
HVO_0318		REG	receiver/sensor/bat box HTH-10 family transcription regulator
HVO_0568		REG	TrmB family transcription regulator
HVO_0633	*cirC*	REG	KaiC-type circadian regulator Circ3
HVO_0815		REG	arNOG05395 family transcription regulator
HVO_1117	*boa2*	REG	HTH-10 family transcription regulator
HVO_1181	*phoU1*	REG	transcription regulator (homolog to phosphate uptake regulator)
HVO_1265	*lrp*	REG	Lrp/AsnC family transcription regulator
HVO_1272		REG	TrmB family transcription regulator
HVO_1299		REG	HTH domain protein
HVO_1357		REG	receiver/bat box HTH-10 family transcription regulator
HVO_1419		REG	HTH-10 family transcription regulator
HVO_1501	*glpR*	REG	DeoR family transcription regulator GlpR
HVO_1552		REG	ArsR family transcription regulator
HVO_1622		REG	ArsR family transcription regulator
HVO_1667		REG	TrmB family transcription regulator
HVO_1752		REG	Lrp/AsnC family transcription regulator
HVO_1792	*trh4*	REG	Lrp/AsnC family transcription regulator
HVO_1929		REG	HTH-10 family transcription regulator
HVO_1953		REG	receiver/sensor/bat box HTH-10 family transcription regulator (nonfunctional)
HVO_2029	*trh5*	REG	Lrp/AsnC family transcription regulator
HVO_2035		REG	TrmB family transcription regulator
HVO_2073		REG	TrmB family transcription regulator
HVO_2108		REG	IclR family transcription regulator
HVO_2130		REG	IclR family transcription regulator
HVO_2349		REG	TrmB family transcription regulator
HVO_2374	*phoU2*	REG	transcription regulator (homolog to phosphate uptake regulator)
HVO_2379	*phoU3*	REG	transcription regulator (homolog to phosphate uptake regulator)
HVO_2412	*trh6*	REG	Lrp/AsnC family transcription regulator
HVO_2421		REG	probable DNA-binding protein
HVO_2507	*trh7*	REG	Lrp/AsnC family transcription regulator
HVO_2688		REG	TrmB family transcription regulator
HVO_2858	*asnC*	REG	Lrp/AsnC family transcription regulator
HVO_2894		REG	HTH-10 family transcription regulator
HVO_A0082		REG	IclR family transcription regulator
HVO_A0093		REG	IclR family transcription regulator
HVO_A0150	*trmB1*	REG	TrmB family transcription regulator
HVO_A0157	*boa4*	REG	integrase family protein / bat box HTH-10 family transcription regulator
HVO_A0168	*boa5*	REG	HTH-10 family transcription regulator
HVO_A0266		REG	IclR family transcription regulator

HVO_A0307	*trh9*	REG	Lrp/AsnC family transcription regulator
HVO_A0332		REG	IclR family transcription regulator
HVO_A0342		REG	IclR family transcription regulator
HVO_A0388		REG	Lrp/AsnC family transcription regulator
HVO_A0465		REG	TetR family transcription regulator
HVO_A0545		REG	TetR family transcription regulator
HVO_A0583		REG	IclR family transcription regulator
HVO_A0589		REG	HxlR family transcription regulator
HVO_B0040		REG	IclR family transcription regulator
HVO_B0066	*trh10*	REG	Lrp/AsnC family transcription regulator
HVO_B0101		REG	IclR family transcription regulator
HVO_B0114		REG	IclR family transcription regulator
HVO_B0119		REG	IclR family transcription regulator
HVO_B0319		REG	IclR family transcription regulator
HVO_B0361		REG	HTH-10 family transcription regulator
HVO_0110		SIG	sensor box histidine kinase
HVO_0204	*kinA1*	SIG	sensor box histidine kinase
HVO_0301		SIG	receiver/sensor box histidine kinase
HVO_0420	*mpcT, htr14*	SIG	transducer protein MpcT
HVO_0554	*basT, htr3*	SIG	transducer protein BasT
HVO_0570	*kinA2*	SIG	histidine kinase
HVO_0621		SIG	sensor box histidine kinase
HVO_0969	*htr39*	SIG	transducer protein Htr39
HVO_1223	*cheA*	SIG	taxis sensor histidine kinase CheA
HVO_1356		SIG	sensor box histidine kinase
HVO_1456		SIG	receiver/sensor box histidine kinase
HVO_1713		SIG	receiver/sensor box histidine kinase
HVO_1779	*htr8*	SIG	transducer protein Htr8
HVO_1793		SIG	receiver/sensor box histidine kinase (nonfunctional)
HVO_1999	*htr7*	SIG	transducer protein Htr7
HVO_2012		SIG	receiver box response regulator
HVO_2195		SIG	sensor box histidine kinase (nonfunctional)
HVO_2214	*htr36*	SIG	transducer protein Htr36
HVO_2306		SIG	receiver box response regulator
HVO_2339	*afsQ*	SIG	sensor box histidine kinase
HVO_2835	*ark3*	SIG	histidine kinase
HVO_2976	*cstA*	SIG	carbon starvation protein CstA
HVO_3005	*htr15B*	SIG	transducer protein Htr15
HVO_A0160	*htlD1*	SIG	sensor box histidine kinase
HVO_A0550		SIG	receiver/sensor box histidine kinase
HVO_A0629	*htlD5*	SIG	sensor box histidine kinase
HVO_B0143	*htlD4*	SIG	receiver/sensor box histidine kinase
HVO_B0154		SIG	receiver/sensor box histidine kinase
HVO_B0196	*kinA8*	SIG	histidine kinase
HVO_B0273	*kinA7*	SIG	sensor box histidine kinase
Genetic information processing			
HVO_0133	*ths1, cct1*	CHP	thermosome subunit 1
HVO_0450	*hsp20A*	CHP	Hsp20-type molecular chaperone
HVO_0455	*ths2, cct2*	CHP	thermosome subunit 2
HVO_0766	*hsp20D*	CHP	Hsp20-type molecular chaperone
HVO_0778	*ths3, cct3*	CHP	thermosome subunit 3
HVO_1589	*dnaJ*	CHP	molecular chaperone DnaJ
HVO_1590	*dnaK*	CHP	DnaK-type molecular chaperone Hsp70
HVO_1592	*grpE*	CHP	DnaJ/DnaK ATPase stimulator GrpE
HVO_2303	*hsp20E*	CHP	Hsp20-type molecular chaperone
HVO_0156	*trmG10*	RMT	tRNA (guanine(10),N(2))-dimethyltransferase
HVO_0180	*rlmE, ftsJ*	RMT	23S rRNA (uridine-2'-O-) methyltransferase
HVO_0236	*trm1*	RMT	tRNA (guanine(26)-N(2))-dimethyltransferase
HVO_0339	*tiaS*	RMT	tRNA(Ile2) 2-agmatinylcytidine synthetase TiaS
HVO_0521	*cca*	RMT	tRNA adenylyltransferase, CCA-adding
HVO_0580	*tuc1*	RMT	putative tRNA 2-thiolation protein
HVO_0658	*truD*	RMT	tRNA pseudouridine(13) synthase TruD
HVO_0929	*trm5, trmM*	RMT	tRNA (guanine(37)-N(1))-methyltransferase
HVO_1094	*rnp3*	RMT	ribonuclease P protein component 3
HVO_1173	*trm56*	RMT	tRNA (cytidine(56)-2'-O)-methyltransferase
HVO_1383	*trmI*	RMT	tRNA (adenine-N(1))-methyltransferase TrmI
HVO_1594	*cna*	RMT	tRNA/rRNA cytosine-C5-methylase
HVO_1669	*fib*	RMT	fibrillarin-like rRNA/tRNA 2'-O-methyltransferase
HVO_1670	*nop5*	RMT	rRNA/tRNA 2'-O-methyltransferase complex protein Nop5

HVO_1716	*queC*	RMT	7-cyano-7-deazaguanine synthase
HVO_1718	*queD*	RMT	6-carboxy-5,6,7,8-tetrahydropterin synthase
HVO_1895	*kae1*	RMT	KEOPS complex subunit Kae1/Bud32
HVO_1965	*nob1*	RMT	rRNA maturation endonuclease Nob1
HVO_1979	*pus10*	RMT	tRNA pseudouridine synthase Pus10
HVO_1989	*trmY*	RMT	tRNA (pseudouridine(54)-N(1))-methyltransferase
HVO_2001	*tgtA*	RMT	tRNA-guanine(15) transglycosylase
HVO_2008	*arcS, tgtA2*	RMT	archaeosine synthase
HVO_2605		RMT	MiaB-like tRNA modifying enzyme
HVO_2712	*rtcB*	RMT	tRNA-splicing ligase RtcB
HVO_2736	*tmcA*	RMT	tRNA(Met) cytidine acetyltransferase
HVO_2746	*ksgA*	RMT	ribosome biogenesis protein KsgA, 16S rRNA-methylating
HVO_2747		RMT	putative tRNA-specific adenosine deaminase
HVO_2906		RMT	tRNA (cytidine/uridine-2'-O-)-methyltransferase
HVO_2952	*endA*	RMT	tRNA-splicing endonuclease
HVO_0003	*polD1*	RRR	DNA-directed DNA polymerase D exonuclease subunit DP1
HVO_0014	*hel308a*	RRR	ATP-dependent DNA helicase Hel308a
HVO_0029	*uvrB*	RRR	UvrABC system protein B
HVO_0039	*rad3b*	RRR	DNA repair helicase Rad3
HVO_0065	*polD2*	RRR	DNA-directed DNA polymerase D large subunit
HVO_0073	*recJ1*	RRR	single-stranded-DNA-specific exonuclease RecJ1
HVO_0104	*radA*	RRR	DNA repair and recombination protein RadA
HVO_0145	*rfcC*	RRR	replication factor C small subunit
HVO_0170	*hjc*	RRR	Holliday junction resolvase
HVO_0173	*priL, priB*	RRR	DNA primase large subunit
HVO_0175	*pcnA*	RRR	DNA polymerase sliding clamp
HVO_0191	*mutS5a*	RRR	DNA mismatch repair protein MutS
HVO_0203	*rfcA*	RRR	replication factor C small subunit
HVO_0220	*mcm*	RRR	ATP-dependent DNA helicase MCM
HVO_0258		RRR	XerC/D-like integrase
HVO_0292	*rpa3*	RRR	replication protein A
HVO_0393	*uvrA*	RRR	UvrABC system protein A
HVO_0415	*uvrD*	RRR	repair helicase
HVO_0519	*rpa2*	RRR	replication protein A
HVO_0551	*mutLb*	RRR	DNA mismatch repair protein MutL
HVO_0552	*mutS1b*	RRR	DNA mismatch repair protein MutS
HVO_0573	*apn1*	RRR	endonuclease IV
HVO_0681	*topA*	RRR	DNA topoisomerase 1
HVO_0732	*rnhE*	RRR	ribonuclease H, type 1
HVO_0741	*polX*	RRR	DNA-directed DNA polymerase X
HVO_0848	*nthA*	RRR	endonuclease III
HVO_0853	*mre11*	RRR	DNA double-strand break repair protein Mre11
HVO_0854	*rad50*	RRR	DNA double-strand break repair ATPase Rad50
HVO_0858	*polB1*	RRR	DNA-directed DNA polymerase B (intein-containing)
HVO_1337	*rpap1*	RRR	rpa-associated protein
HVO_1338	*rpa1*	RRR	replication protein A
HVO_1351	*rad3a*	RRR	DNA repair helicase Rad3
HVO_1354	*mutS5b*	RRR	DNA mismatch repair protein MutS
HVO_1565	*ligA*	RRR	DNA ligase (ATP)
HVO_1570	*top6A*	RRR	DNA topoisomerase 6 subunit A
HVO_1571	*top6B*	RRR	DNA topoisomerase 6 subunit B
HVO_1572	*gyrB*	RRR	DNA gyrase subunit B
HVO_1573	*gyrA*	RRR	DNA gyrase subunit A
HVO_1723	*rad25d*	RRR	DNA repair helicase Rad25
HVO_1839		RRR	XerC/D-like integrase
HVO_1940	*mutS1a*	RRR	DNA mismatch repair protein MutS
HVO_2321	*dnaG*	RRR	DNA primase DnaG
HVO_2383	*radB*	RRR	DNA repair and recombination protein RadB
HVO_2427	*rfcB*	RRR	replication factor C large subunit
HVO_2697	*priS, priA*	RRR	DNA primase small subunit
HVO_2767	*dna2*	RRR	ATP-dependent DNA helicase Dna2
HVO_2827		RRR	UvrD/REP family helicase
HVO_2873	*fen1*	RRR	flap endonuclease Fen1
HVO_2911	*phr1*	RRR	deoxyribodipyrimidine photolyase
HVO_3000	*ligN*	RRR	DNA ligase (NAD)
HVO_3006	*uvrC*	RRR	UvrABC system protein C
HVO_A0126		RRR	XerC/D-like integrase
HVO_A0277	*rnhD*	RRR	ribonuclease H, type 1 (nonfunctional)
HVO_A0346		RRR	XerC/D-like integrase
HVO_0158	*tbp1*	TC	TATA-binding transcription initiation factor

HVO_0347	*rpoB2*	TC	DNA-directed RNA polymerase subunit B''
HVO_0348	*rpoB1*	TC	DNA-directed RNA polymerase subunit B'
HVO_0349	*rpoA1*	TC	DNA-directed RNA polymerase subunit A'
HVO_0350	*rpoA2*	TC	DNA-directed RNA polymerase subunit A''
HVO_0351	*nusA*	TC	transcription elongation factor NusA
HVO_0719	*spt5, nusG*	TC	transcription elongation factor Spt5
HVO_0733	*tfb4*	TC	transcription initiation factor TFB
HVO_0795	*tfb6*	TC	transcription initiation factor TFB
HVO_1052	*tfb1*	TC	transcription initiation factor TFB
HVO_1174	*tfeA*	TC	transcription initiation factor TFE
HVO_1478	*tfb5*	TC	transcription initiation factor TFB
HVO_1676	*tfb2*	TC	transcription initiation factor TFB
HVO_1727	*tbp2*	TC	TATA-binding transcription initiation factor
HVO_1899	*rpoE1*	TC	DNA-directed RNA polymerase subunit E
HVO_2781	*rpoD*	TC	DNA-directed RNA polymerase subunit D
HVO_B0285	*tfb3*	TC	transcription initiation factor TFB
HVO_B0382	*tbp3*	TC	TATA-binding transcription initiation factor
HVO_0054	*glyS*	TL	glycine--tRNA ligase
HVO_0115	*rpl39e*	TL	50S ribosomal protein L39e
HVO_0116	*rpl31e*	TL	50S ribosomal protein L31e
HVO_0117	*tif6*	TL	translation initiation factor aIF-6
HVO_0118	*rpl20e, rplX*	TL	50S ribosomal protein L20e
HVO_0138	*tyrS*	TL	tyrosine--tRNA ligase
HVO_0206	*alaS1*	TL	alanine--tRNA ligase
HVO_0321	*arf1*	TL	peptide chain release factor aRF-1
HVO_0324	*argS*	TL	arginine--tRNA ligase
HVO_0353	*rps12*	TL	30S ribosomal protein S12
HVO_0354	*rps7*	TL	30S ribosomal protein S7
HVO_0356	*tef2*	TL	translation elongation factor aEF-2
HVO_0359	*tef1a1*	TL	translation elongation factor aEF-1 alpha subunit
HVO_0360	*rps10a*	TL	30S ribosomal protein S10a
HVO_0452	*leuS*	TL	leucine--tRNA ligase
HVO_0484	*rpl16*	TL	50S ribosomal protein L16
HVO_0561	*rpl15e*	TL	50S ribosomal protein L15e
HVO_0654	*rpl43e*	TL	50S ribosomal protein L43e
HVO_0677	*aspS*	TL	aspartate--tRNA ligase
HVO_0684	*gatB, aatB*	TL	aspartyl/glutamyl-tRNA(Asn/Gln) amidotransferase subunit B
HVO_0699	*tif2a*	TL	translation initiation factor aIF-2 alpha subunit
HVO_0809	*metS*	TL	methionine--tRNA ligase
HVO_0870	*proS*	TL	proline--tRNA ligase
HVO_1054	*gatA, aatA*	TL	aspartyl/glutamyl-tRNA(Asn/Gln) amidotransferase subunit A
HVO_1125	*cysS*	TL	cysteine--tRNA ligase
HVO_1145	*rps1e*	TL	30S ribosomal protein S1e
HVO_1148	*rps15*	TL	30S ribosomal protein S15
HVO_1382	*nac*	TL	nascent polypeptide-associated complex protein
HVO_1547	*ileS*	TL	isoleucine--tRNA ligase
HVO_1684	*thrS*	TL	threonine--tRNA ligase
HVO_1827	*rps6e*	TL	30S ribosomal protein S6e
HVO_1854	*hisS*	TL	histidine--tRNA ligase
HVO_1858	*rps19e*	TL	30S ribosomal protein S19e
HVO_1867	*lysS*	TL	lysine--tRNA ligase
HVO_1896	*rps24e*	TL	30S ribosomal protein S24e
HVO_1901	*tif2c*	TL	translation initiation factor aIF-2 gamma subunit
HVO_1921	*serS*	TL	serine--tRNA ligase
HVO_1934	*tif2Ba*	TL	translation initiation factor aIF-2B alpha subunit
HVO_1946	*tif1a*	TL	translation initiation factor aIF-1 (SUI1 protein, bacterial-type IF3)
HVO_1963	*tif5B*	TL	translation initiation factor aIF-5B (bacterial-type IF2)
HVO_2242		TL	homolog to translation initiation factor aIF-2 beta subunit / probable RNA-binding protein
HVO_2300	*tif5A*	TL	translation initiation factor aIF-5A
HVO_2373	*rps8e*	TL	30S ribosomal protein S8e
HVO_2413	*tef1a2*	TL	translation elongation factor aEF-1 alpha subunit
HVO_2511	*gatD*	TL	glutamyl-tRNA(Gln) amidotransferase subunit D
HVO_2542	*rpl15*	TL	50S ribosomal protein L15
HVO_2543	*rpl30*	TL	50S ribosomal protein L30
HVO_2544	*rps5*	TL	30S ribosomal protein S5
HVO_2545	*rpl18*	TL	50S ribosomal protein L18
HVO_2546	*rpl19e*	TL	50S ribosomal protein L19e
HVO_2547	*rpl32e*	TL	50S ribosomal protein L32e
HVO_2548	*rpl6*	TL	50S ribosomal protein L6
HVO_2549	*rps8*	TL	30S ribosomal protein S8

HVO_2551	*rpl5*	TL	50S ribosomal protein L5
HVO_2552	*rps4e*	TL	30S ribosomal protein S4e
HVO_2553	*rpl24*	TL	50S ribosomal protein L24
HVO_2554	*rpl14*	TL	50S ribosomal protein L14
HVO_2555	*rps17*	TL	30S ribosomal protein S17
HVO_2557	*rpl29*	TL	50S ribosomal protein L29
HVO_2558	*rps3*	TL	30S ribosomal protein S3
HVO_2559	*rpl22*	TL	50S ribosomal protein L22
HVO_2560	*rps19*	TL	30S ribosomal protein S19
HVO_2561	*rpl2*	TL	50S ribosomal protein L2
HVO_2562	*rpl23*	TL	50S ribosomal protein L23
HVO_2563	*rpl4*	TL	50S ribosomal protein L4
HVO_2564	*rpl3*	TL	50S ribosomal protein L3
HVO_2706	*tif2Bd*	TL	translation initiation factor aIF-2B delta subunit
HVO_2717	*alaS2*	TL	alanine--tRNA ligase
HVO_2726	*gltS*	TL	glutamate--tRNA ligase
HVO_2737	*rpl8e*	TL	50S ribosomal protein L8e
HVO_2739	*rpl24e*	TL	50S ribosomal protein L24e
HVO_2749	*rpl21e*	TL	50S ribosomal protein L21e
HVO_2756	*rpl10*	TL	50S ribosomal protein L10
HVO_2757	*rpl1*	TL	50S ribosomal protein L1
HVO_2758	*rpl11*	TL	50S ribosomal protein L11
HVO_2773	*rps2*	TL	30S ribosomal protein S2
HVO_2777	*rps9*	TL	30S ribosomal protein S9
HVO_2778	*rpl13*	TL	50S ribosomal protein L13
HVO_2779	*rpl18e*	TL	50S ribosomal protein L18e
HVO_2782	*rps11*	TL	30S ribosomal protein S11
HVO_2783	*rps4*	TL	30S ribosomal protein S4
HVO_2784	*rps13*	TL	30S ribosomal protein S13
HVO_2880	*pelA*	TL	probable peptide chain release factor pelota
HVO_2902	*gatE*	TL	glutamyl-tRNA(Gln) amidotransferase subunit E
HVO_2945	*valS*	TL	valine--tRNA ligase
HVO_2947	*pheT*	TL	phenylalanine--tRNA ligase beta subunit
HVO_2948	*pheS*	TL	phenylalanine--tRNA ligase alpha subunit
HVO_2951	*trpS1*	TL	tryptophan--tRNA ligase
HVO_A0127	*trpS2*	TL	tryptophan--tRNA ligase
Metabolism			
HVO_0008	*lysC*	AA	aspartate kinase
HVO_0009	*tnaA*	AA	tryptophanase
HVO_0041	*argF*	AA	ornithine carbamoyltransferase
HVO_0042	*argE*	AA	acetylornithine deacetylase
HVO_0043	*argD*	AA	acetylornithine aminotransferase
HVO_0044	*argB*	AA	acetylglutamate kinase
HVO_0045	*argC*	AA	N-acetyl-gamma-glutamyl-phosphate reductase
HVO_0046	*argX*	AA	putative glutamate--argW ligase
HVO_0048	*argH*	AA	argininosuccinate lyase
HVO_0049	*argG*	AA	argininosuccinate synthase
HVO_0109	*sufS1*	AA	cysteine desulfurase
HVO_0122	*serA1*	AA	phosphoglycerate dehydrogenase
HVO_0148	*ureC*	AA	urease alpha subunit
HVO_0150	*ureG*	AA	urease accessory protein UreG
HVO_0151	*ureD*	AA	urease accessory protein UreD
HVO_0153	*ureF*	AA	urease accessory protein UreF
HVO_0161	*hisG*	AA	ATP phosphoribosyltransferase
HVO_0167	*ahcY*	AA	adenosylhomocysteinase
HVO_0239	*glnA*	AA	glutamine synthetase
HVO_0243	*lta*	AA	threonine aldolase
HVO_0329	*ilvE*	AA	branched-chain amino acid aminotransferase
HVO_0331	*fadM1*	AA	proline dehydrogenase
HVO_0358	*hom1*	AA	homoserine dehydrogenase
HVO_0448	*hisH*	AA	imidazoleglycerol-phosphate synthase subunit HisH
HVO_0449	*pheA*	AA	prephenate dehydratase
HVO_0454	*ala*	AA	alanine dehydrogenase
HVO_0464	*ilvA1*	AA	threonine ammonia-lyase
HVO_0470	*thrC1*	AA	threonine synthase
HVO_0507	*cre1*	AA	creatininase
HVO_0601	*hom2*	AA	homoserine dehydrogenase
HVO_0602	*aroD1*	AA	3-dehydroquinate dehydratase
HVO_0644	*leuA1, cimA*	AA	2-isopropylmalate synthase / (R)-citramalate synthase
HVO_0712	*aroE*	AA	shikimate dehydrogenase (nonfunctional)

HVO_0787	*trpC*	AA	indole-3-glycerol-phosphate synthase
HVO_0788	*trpB*	AA	tryptophan synthase beta subunit
HVO_0789	*trpA*	AA	tryptophan synthase alpha subunit
HVO_0790	*fba2*	AA	2-amino-3,7-dideoxy-D-threo-hept-6-ulosonate synthase
HVO_0792	*aroB*	AA	3-dehydroquinate synthase type II
HVO_0811	*mfnA*	AA	tyrosine decarboxylase
HVO_0818	*thrC2*	AA	threonine synthase
HVO_0869	*gltB*	AA	glutamate synthase large subunit
HVO_1044	*hisF*	AA	imidazoleglycerol-phosphate synthase subunit HisF
HVO_1079	*cysD1*	AA	sulfate adenylyltransferase small subunit
HVO_1096	*dapE*	AA	succinyl-diaminopimelate desuccinylase
HVO_1097	*dapF*	AA	diaminopimelate epimerase
HVO_1098	*lysA*	AA	diaminopimelate decarboxylase
HVO_1099	*dapD*	AA	2,3,4,5-tetrahydropyridine-2,6-dicarboxylate N-succinyltransferase
HVO_1100	*dapB*	AA	4-hydroxy-tetrahydrodipicolinate reductase
HVO_1101	*dapA*	AA	4-hydroxy-tetrahydrodipicolinate synthase
HVO_1191	*fadM2*	AA	proline dehydrogenase
HVO_1235	*cre2*	AA	creatininase
HVO_1295	*hisC*	AA	histidinol-phosphate aminotransferase
HVO_1306	*aroC*	AA	chorismate synthase
HVO_1308	*aroA*	AA	3-phosphoshikimate 1-carboxyvinyltransferase
HVO_1323	*aroK*	AA	shikimate kinase, archaeal-type
HVO_1370	*proA*	AA	gamma-glutamyl phosphate reductase
HVO_1371	*proB*	AA	glutamate 5-kinase
HVO_1372	*proC*	AA	pyrroline-5-carboxylate reductase
HVO_1439	*cysK1*	AA	cysteine synthase
HVO_1453	*gdhA2*	AA	glutamate dehydrogenase (NADP)
HVO_1502	*leuB*	AA	3-isopropylmalate dehydrogenase
HVO_1503	*leuD1*	AA	3-isopropylmalate dehydratase small subunit
HVO_1504	*leuC1*	AA	3-isopropylmalate dehydratase large subunit
HVO_1506	*ilvC*	AA	ketol-acid reductoisomerase
HVO_1507	*ilvN*	AA	acetolactate synthase small subunit
HVO_1508	*ilvB1*	AA	acetolactate synthase large subunit
HVO_1510	*leuA2*	AA	2-isopropylmalate synthase
HVO_1553		AA	hydroxypyruvate reductase
HVO_1575	*rocF*	AA	arginase
HVO_1654	*cysK2*	AA	cysteine synthase
HVO_2004	*cysE*	AA	serine O-acetyltransferase
HVO_2209	*oadhA4*	AA	probable branched-chain amino acid dehydrogenase E1 component alpha subunit
HVO_2338	*thrB*	AA	homoserine kinase
HVO_2361	*carB*	AA	carbamoyl-phosphate synthase (glutamine-hydrolyzing) large subunit
HVO_2401	*gcvP2*	AA	glycine cleavage system protein P beta subunit
HVO_2402	*gcvP1*	AA	glycine cleavage system protein P alpha subunit
HVO_2404	*gcvT*	AA	glycine cleavage system protein T
HVO_2453	*trpG*	AA	anthranilate synthase component 2
HVO_2454	*trpE*	AA	anthranilate synthase component 1
HVO_2456	*trpD1*	AA	anthranilate phosphoribosyltransferase
HVO_2487	*asd*	AA	aspartate-semialdehyde dehydrogenase
HVO_2503	*kynU*	AA	kynureninase
HVO_2508	*carA*	AA	carbamoyl-phosphate synthase (glutamine-hydrolyzing) small subunit
HVO_2646	*ilvD*	AA	dihydroxy-acid dehydratase
HVO_2648	*serA2*	AA	phosphoglycerate dehydrogenase
HVO_2675	*hisD*	AA	histidinol dehydrogenase
HVO_2742	*metE1*	AA	5-methyltetrahydropteroyltriglutamate--homocysteine S-methyltransferase (methionine synthase II)
HVO_2743	*metE2*	AA	5-methyltetrahydropteroyltriglutamate--homocysteine S-methyltransferase (methionine synthase II)
HVO_2750	*metB1*	AA	cystathionine synthase/lyase (cystathionine gamma-synthase, cystathionine gamma-lyase, cystathionine beta-lyase)
HVO_2862	*glyA1*	AA	serine hydroxymethyltransferase
HVO_2879	*ocd2*	AA	ornithine cyclodeaminase
HVO_2946	*metB2*	AA	cystathionine synthase/lyase (cystathionine gamma-synthase, cystathionine gamma-lyase, cystathionine beta-lyase)
HVO_2958	*oadhA1*	AA	2-oxo-3-methylvalerate dehydrogenase E1 component alpha subunit
HVO_2959	*oadhB1*	AA	2-oxo-3-methylvalerate dehydrogenase E1 component beta subunit
HVO_2965	*serB*	AA	phosphoserine phosphatase
HVO_2968	*serA3*	AA	phosphoglycerate dehydrogenase
HVO_2969	*thrC3*	AA	threonine synthase
HVO_2986	*hisB*	AA	imidazoleglycerol-phosphate dehydratase
HVO_2988	*hisA*	AA	1-(5-phosphoribosyl)-5-[(5-phosphoribosylamino)methylideneamino] imidazole-4-carboxamide isomerase
HVO_2997	*metY2*	AA	O-acetylhomoserine aminocarboxypropyltransferase (methionine synthase)
HVO_2998	*metX*	AA	homoserine O-acetyltransferase
HVO_2999	*metY1*	AA	O-acetylhomoserine aminocarboxypropyltransferase (methionine synthase)
HVO_A0163	*prr*	AA	proline racemase

HVO_A0306	*gabT6*	AA	4-aminobutyrate aminotransferase
HVO_A0559	*hutH*	AA	histidine ammonia-lyase
HVO_A0560	*hutI*	AA	imidazolonepropionase
HVO_A0561	*hutG*	AA	formimidoylglutamase
HVO_A0562	*hutU*	AA	urocanate hydratase
HVO_A0635	*sufS2*	AA	probable cysteine desulfurase
HVO_B0004	*glyA2*	AA	serine hydroxymethyltransferase
HVO_B0011	*soxA1*	AA	sarcosine oxidase
HVO_B0257	*gabT5*	AA	4-aminobutyrate aminotransferase
HVO_B0264	*serA4*	AA	phosphoglycerate dehydrogenase
HVO_B0266	*gdhA3, gdhB*	AA	glutamate dehydrogenase (NAD)
HVO_C0077	*soxA2*	AA	sarcosine oxidase
HVO_0199	*pmm2*	CHM	phosphohexomutase (phosphoglucomutase / phosphomannomutase)
HVO_0567	*amyA1*	CHM	alpha amylase
HVO_1076	*graD1*	CHM	sugar nucleotidyltransferase
HVO_1083	*gdh*	CHM	glucose 1-dehydrogenase
HVO_1348		CHM	probable YjeF family carbohydrate kinase
HVO_1402	*pmm3*	CHM	phosphohexomutase (phosphoglucomutase / phosphomannomutase)
HVO_1488	*gnaD*	CHM	D-gluconate dehydratase
HVO_1494	*fba1*	CHM	fructose-1,6-bisphosphate aldolase, class II
HVO_1500	*pfkB*	CHM	1-phosphofructokinase
HVO_1527	*aglF*	CHM	glucose-1-phosphate uridylyltransferase
HVO_1531	*aglM*	CHM	UDP-glucose 6-dehydrogenase
HVO_1683	*malQ*	CHM	4-alpha-glucanotransferase
HVO_1710	*amyA2*	CHM	alpha amylase
HVO_1711	*sga1*	CHM	glucoamylase
HVO_2056	*spsK*	CHM	probable epimerase (homolog to dTDP-4-dehydrorhamnose 3,5-epimerase)
HVO_2057	*graD2*	CHM	sugar nucleotidyltransferase
HVO_2058		CHM	NAD-dependent epimerase/dehydratase (homolog to dTDP-4-dehydrorhamnose reductase)
HVO_2059	*galE5*	CHM	NAD-dependent epimerase/dehydratase
HVO_2989	*pmm4*	CHM	phosphohexomutase (phosphoglucomutase / phosphomannomutase)
HVO_A0267	*dgoD2*	CHM	D-galactonate dehydratase
HVO_A0331	*dgoD1*	CHM	D-galactonate dehydratase
HVO_A0586	*graD3*	CHM	sugar nucleotidyltransferase
HVO_B0027		CHM	2-keto-3-deoxyxylonate dehydratase
HVO_B0029	*xdh2*	CHM	xylose dehydrogenase (NADP)
HVO_B0038A	*xad*	CHM	D-xylonate dehydratase
HVO_B0039		CHM	alpha-ketoglutarate semialdehyde dehydrogenase
HVO_B0233	*xsa*	CHM	alpha-L-arabinofuranosidase
HVO_0195	*rpiA*	CIM	ribose-5-phosphate isomerase
HVO_0214		CIM	L-lactate dehydrogenase
HVO_0233	*prsA*	CIM	ribose-phosphate pyrophosphokinase
HVO_0466	*citZ*	CIM	citrate (si)-synthase
HVO_0478	*gap1*	CIM	glyceraldehyde-3-phosphate dehydrogenase (NAD(P)) (phosphorylating)
HVO_0480	*pgk*	CIM	phosphoglycerate kinase
HVO_0481	*gap2*	CIM	glyceraldehyde-3-phosphate dehydrogenase (NAD) (phosphorylating)
HVO_0541	*citB2, acnA*	CIM	aconitate hydratase
HVO_0549	*kdgK1*	CIM	2-keto-3-deoxygluconate kinase
HVO_0806	*pykA*	CIM	pyruvate kinase
HVO_0812	*ppsA*	CIM	phosphoenolpyruvate synthase
HVO_0822	*gldA*	CIM	glycerol-1-phosphate dehydrogenase [NAD(P)]
HVO_0887	*korB*	CIM	oxoglutarate--ferredoxin oxidoreductase beta subunit
HVO_0888	*korA*	CIM	oxoglutarate--ferredoxin oxidoreductase alpha subunit
HVO_0893	*mmcA1*	CIM	methylmalonyl-CoA mutase subunit A
HVO_0950	*kdgA1*	CIM	2-dehydro-3-deoxy-phosphogluconate aldolase, bacterial-type
HVO_0966		CIM	ribose-1,5-bisphosphate isomerase (ribulose-bisphosphate forming)
HVO_0970	*rbcL*	CIM	ribulose-bisphosphate carboxylase large subunit
HVO_1300	*tpiA1*	CIM	triosephosphate isomerase
HVO_1304	*porB*	CIM	pyruvate--ferredoxin oxidoreductase beta subunit
HVO_1305	*porA*	CIM	pyruvate--ferredoxin oxidoreductase alpha subunit
HVO_1380	*mmcA2*	CIM	methylmalonyl-CoA mutase subunit A
HVO_1446	*fbp1*	CIM	fructose-1,6-bisphosphatase
HVO_1452	*citE*	CIM	citryl-CoA lyase (citrate lyase beta subunit / ATP citrate synthase beta subunit)
HVO_1496	*ptsI*	CIM	phosphotransferase system component I
HVO_1536		CIM	phosphoglycolate phosphatase
HVO_1538	*glpA1*	CIM	glycerol-3-phosphate dehydrogenase subunit A
HVO_1539	*glpB1*	CIM	glycerol-3-phosphate dehydrogenase subunit B
HVO_1540	*glpC1*	CIM	glycerol-3-phosphate dehydrogenase subunit C
HVO_1541	*glpK*	CIM	glycerol kinase

HVO_1545	*dhaL*	CIM	dihydroxyacetone kinase subunit DhaL
HVO_1546	*dhaK*	CIM	dihydroxyacetone kinase subunit DhaK
HVO_1830	*gndA*	CIM	6-phosphogluconate dehydrogenase (decarboxylating)
HVO_1955	*citB1*	CIM	aconitate hydratase
HVO_1967	*pgi*	CIM	glucose-6-phosphate isomerase
HVO_1983	*aceB1*	CIM	malate synthase
HVO_2131	*fbp2*	CIM	fructose-1,6-bisphosphatase
HVO_2158	*maeB1, mdh1*	CIM	malic enzyme (NADP)
HVO_2436	*maeB2, mdh2*	CIM	malic enzyme (NADP)
HVO_2464	*sucD*	CIM	succinate--CoA ligase (ADP-forming) alpha subunit
HVO_2465	*sucC*	CIM	succinate--CoA ligase (ADP-forming) beta subunit
HVO_2486	*pccA*	CIM	propionyl-CoA carboxylase biotin carboxylase component
HVO_2516	*gpmI*	CIM	phosphoglycerate mutase, 2,3-biphosphateglycerate-independent type
HVO_2588	*icd*	CIM	isocitrate dehydrogenase (NADP)
HVO_2610	*deoC*	CIM	deoxyribose-phosphate aldolase
HVO_2621	*ppc*	CIM	phosphoenolpyruvate carboxylase
HVO_2774	*eno*	CIM	enolase
HVO_2808	*sdhA*	CIM	succinate dehydrogenase subunit A
HVO_2809	*sdhB*	CIM	succinate dehydrogenase subunit B
HVO_2810	*sdhD*	CIM	succinate dehydrogenase subunit D
HVO_2857	*suhB1*	CIM	probable inositol-1(or 4)-monophosphatase / fructose-1,6-bisphosphatase, archaeal-type
HVO_2900	*fumC*	CIM	fumarate hydratase
HVO_2961	*lpdA*	CIM	dihydrolipoyl dehydrogenase
HVO_3007	*mdh*	CIM	malate dehydrogenase
HVO_A0269	*glpA2*	CIM	glycerol-3-phosphate dehydrogenase subunit A
HVO_A0270	*glpB2*	CIM	glycerol-3-phosphate dehydrogenase subunit B
HVO_A0271	*glpC2*	CIM	glycerol-3-phosphate dehydrogenase subunit C
HVO_A0274	*suhB2*	CIM	probable inositol-1(or 4)-monophosphatase / fructose-1,6-bisphosphatase, archaeal-type
HVO_A0305	*mmsA*	CIM	methylmalonate-semialdehyde dehydrogenase
HVO_A0328	*kdgK2*	CIM	2-keto-3-deoxygluconate kinase
HVO_A0329	*kdgA3*	CIM	2-dehydro-3-deoxy-phosphogluconate aldolase, bacterial-type
HVO_B0200	*aceB2*	CIM	malate synthase
HVO_B0253	*suhB3*	CIM	probable inositol-1(or 4)-monophosphatase / fructose-1,6-bisphosphatase, archaeal-type
HVO_0076	*hemD*	COM	uroporphyrinogen-III synthase
HVO_0077	*sirA*	COM	uroporphyrin-III C-methyltransferase
HVO_0078	*hemC*	COM	hydroxymethylbilane synthase (porphobilinogen deaminase)
HVO_0081	*hemL*	COM	glutamate-1-semialdehyde 2,1-aminomutase
HVO_0087	*hemB*	COM	porphobilinogen synthase
HVO_0309	*hmp*	COM	S-adenosylmethionine-dependent methyltransferase
HVO_0326	*ribK, rfk*	COM	CTP-dependent riboflavin kinase
HVO_0409	*ribE*	COM	riboflavin synthase
HVO_0588	*cobS*	COM	cobalamin (5'-phosphate) synthase
HVO_0590	*cobT*	COM	nicotinate-nucleotide-dimethylbenzimidazole phosphoribosyltransferase
HVO_0591	*cobD1*	COM	L-threonine-O-3-phosphate decarboxylase
HVO_0662	*thiN*	COM	HTH domain protein / thiamine-phosphate synthase
HVO_0675		COM	pantoate kinase
HVO_0708	*pabC*	COM	aminodeoxychorismate lyase
HVO_0710	*pabB*	COM	aminodeoxychorismate synthase component 1
HVO_0837	*ppnK2*	COM	probable NAD kinase (inorganic polyphosphate/ATP NAD kinase)
HVO_0974	*ribH*	COM	6,7-dimethyl-8-ribityllumazine synthase
HVO_1013	*ctaB*	COM	protoheme IX geranylgeranyltransferase
HVO_1062	*coaBC*	COM	phosphopantothenoylcysteine decarboxylase / phosphopantothenate--cysteine ligase
HVO_1088	*folCP*	COM	folylpolyglutamate synthase / 7,8-dihydropteroate reductase / dihydropteroate synthase
HVO_1121	*ahbC, pqqE1*	COM	Fe-coproporphyrin synthase AhbC
HVO_1128	*cbiX2*	COM	sirohydrochlorin cobaltochelatase
HVO_1279	*hdrA, folA1*	COM	dihydrofolate reductase
HVO_1284	*gch3*	COM	GTP cyclohydrolase III
HVO_1341	*ribG*	COM	2,5-diamino-6-(ribosylamino)-4(3H)-pyrimidinone 5'-phosphate reductase
HVO_1347	*moaC*	COM	probable cyclic pyranopterin monophosphate synthase accessory protein
HVO_1375	*menE*	COM	O-succinylbenzoic acid--CoA ligase
HVO_1462	*menA*	COM	1,4-dihydroxy-2-naphthoate octaprenyltransferase
HVO_1465	*menB*	COM	naphthoate synthase
HVO_1469	*menD*	COM	2-succinyl-5-enolpyruvyl-6-hydroxy-3-cyclohexene-1-carboxylate synthase
HVO_1470	*menF*	COM	isochorismate synthase
HVO_1651	*thiI*	COM	thiamine / thiouridine biosynthesis protein ThiI
HVO_1804	*pncB*	COM	nicotinate phosphoribosyltransferase
HVO_1861	*thiL*	COM	thiamine-monophosphate kinase

HVO_1864	*moaE*	COM	molybdopterin synthase catalytic subunit
HVO_1878	*nadE*	COM	NAD synthase
HVO_1928		COM	5-formyltetrahydrofolate cyclo-ligase
HVO_1936	*cofE*	COM	F420-0:gamma-glutamyl ligase
HVO_2198	*cofH*	COM	7,8-didemethyl-8-hydroxy-5-deazariboflavin synthase subunit 2
HVO_2201	*cofG*	COM	7,8-didemethyl-8-hydroxy-5-deazariboflavin synthase subunit 1
HVO_2227	*ahbA, nirDL*	COM	siroheme decarboxylase AhbA
HVO_2298		COM	UPF0135 family protein
HVO_2302	*ubiB*	COM	ubiquinone biosynthesis protein UbiB
HVO_2304	*moeA1*	COM	molybdenum cofactor biosynthesis protein MoeA
HVO_2305	*moeA2*	COM	molybdenum cofactor biosynthesis protein MoeA
HVO_2309	*cad*	COM	probable pterin-4-alpha-carbinolamine dehydratase
HVO_2311	*hemA*	COM	glutamyl-tRNA reductase
HVO_2313	*ahbB, nirGH*	COM	siroheme decarboxylase AhbB
HVO_2336	*pdxS*	COM	pyridoxal biosynthesis lyase PdxS
HVO_2348	*mptA, folE2*	COM	GTP cyclohydrolase MptA
HVO_2363	*ppnK1*	COM	probable NAD kinase (inorganic polyphosphate/ATP NAD kinase)
HVO_2479	*cofD*	COM	2-phospho-L-lactate transferase
HVO_2573	*mch*	COM	probable methenyltetrahydrofolate cyclohydrolase
HVO_2579	*nadC*	COM	nicotinate-nucleotide pyrophosphorylase (carboxylating)
HVO_2580	*nadB*	COM	L-aspartate oxidase
HVO_2581	*nadA*	COM	quinolinate synthase A
HVO_2703	*panB2*	COM	3-methyl-2-oxobutanoate hydroxymethyltransferase
HVO_2865	*folD*	COM	methylenetetrahydrofolate dehydrogenase / methenyltetrahydrofolate cyclohydrolase
HVO_2908	*folP2, dchpS*	COM	dihydropteroate synthase
HVO_A0487	*cbiA, cobB*	COM	cobyrinic acid a,c-diamide synthase
HVO_A0553	*cbiP, cobQ*	COM	adenosylcobyric acid synthase
HVO_B0048	*cbiE*	COM	precorrin-6Y C5-methyltransferase
HVO_B0049	*cbiC*	COM	precorrin-8 methylmutase
HVO_B0050	*cobN*	COM	ATP-dependent cobaltochelatase subunit CobN
HVO_B0051	*chlID*	COM	ATP-dependent cobaltochelatase subunit ChlID
HVO_B0054	*cbiX1*	COM	sirohydrochlorin cobaltochelatase
HVO_B0057	*cbiH2, cobJ2*	COM	precorrin-3B C17-methyltransferase
HVO_B0058	*cbiH1, cobJ1*	COM	precorrin-3B C17-methyltransferase
HVO_B0059	*cbiG*	COM	cobalt-precorrin 5A hydrolase
HVO_B0060	*cbiF*	COM	precorrin-4 C11-methyltransferase
HVO_B0061	*cbiL*	COM	precorrin-2 C20-methyltransferase
HVO_B0062	*cbiT*	COM	precorrin-7 15-methyltransferase (decarboxylating)
HVO_0304	*etfA1*	EM	electron transfer flavoprotein alpha subunit
HVO_0305	*etfB1*	EM	electron transfer flavoprotein beta subunit
HVO_0310	*atpH*	EM	A-type ATP synthase subunit H
HVO_0311	*atpI*	EM	A-type ATP synthase subunit I
HVO_0313	*atpE*	EM	A-type ATP synthase subunit E
HVO_0314	*atpC*	EM	A-type ATP synthase subunit C
HVO_0316	*atpA*	EM	A-type ATP synthase subunit A
HVO_0317	*atpB*	EM	A-type ATP synthase subunit B
HVO_0319	*atpD1*	EM	A-type ATP synthase subunit D
HVO_0462	*cydA*	EM	cytochrome bd ubiquinol oxidase subunit I
HVO_0841	*petD*	EM	cytochrome bc1 complex cytochrome b/c subunit
HVO_0842	*petB*	EM	cytochrome bc1 complex cytochrome b subunit
HVO_0907	*coxA1*	EM	cox-type terminal oxidase subunit I
HVO_0944	*cbaB*	EM	ba3-type terminal oxidase subunit II
HVO_0945	*cbaA*	EM	ba3-type terminal oxidase subunit I
HVO_0968	*nuoCD2*	EM	NADH dehydrogenase-like complex subunit CD
HVO_0979	*nuoB*	EM	NADH dehydrogenase-like complex subunit B
HVO_0980	*nuoCD1*	EM	NADH dehydrogenase-like complex subunit CD
HVO_0981	*nuoH*	EM	NADH dehydrogenase-like complex subunit H
HVO_0982	*nuoI*	EM	NADH dehydrogenase-like complex subunit I
HVO_0984	*nuoJ2*	EM	NADH dehydrogenase-like complex subunit J2
HVO_0986	*nuoL*	EM	NADH dehydrogenase-like complex subunit L
HVO_0987	*nuoM*	EM	NADH dehydrogenase-like complex subunit M
HVO_0988	*nuoN*	EM	NADH dehydrogenase-like complex subunit N
HVO_1014	*coxB1*	EM	cox-type terminal oxidase subunit II
HVO_1138	*coxC1*	EM	cox-type terminal oxidase subunit III
HVO_1413	*ndh2*	EM	probable NADH dehydrogenase
HVO_1578	*ndh1*	EM	probable NADH dehydrogenase

HVO_1831	*ferA4*	EM	ferredoxin (2Fe-2S)
HVO_2150	*hcpG*	EM	halocyanin
HVO_2620	*petA*	EM	cytochrome bc1 complex Rieske iron-sulfur protein
HVO_2995	*fdx, ferA5*	EM	ferredoxin (2Fe-2S)
HVO_B0164	*narG*	EM	respiratory nitrate reductase catalytic subunit
HVO_B0309	*coxL*	EM	aerobic-type carbon monoxide dehydrogenase large subunit
HVO_B0363	*dmsA*	EM	dimethylsulfoxide reductase subunit A
HVO_B0364	*dmsB*	EM	dimethylsulfoxide reductase subunit B
HVO_0146	*psd*	LIP	phosphatidylserine decarboxylase
HVO_0209	*acd1*	LIP	acyl-CoA dehydrogenase
HVO_0303	*idsA2*	LIP	bifunctional short chain isoprenyl diphosphate synthase
HVO_0680	*pcrB1*	LIP	(S)-3-O-geranylgeranylglyceryl phosphate synthase 1
HVO_0817	*crtI3*	LIP	phytoene dehydrogenase (phytoene desaturase)
HVO_0835	*acaB1*	LIP	acetyl-CoA C-acetyltransferase
HVO_0894	*acs1*	LIP	acyl-CoA synthetase
HVO_0896	*acs2*	LIP	acyl-CoA synthetase
HVO_1000	*acdA*	LIP	acyl-CoA synthetase (homolog to acetate--CoA ligase (ADP-forming))
HVO_1025	*acaB2*	LIP	acetyl-CoA C-acyltransferase
HVO_1139	*fdfT*	LIP	squalene synthase
HVO_1140	*acd2*	LIP	acyl-CoA dehydrogenase
HVO_1199	*acd3*	LIP	acyl-CoA dehydrogenase
HVO_1236	*acs3*	LIP	acyl-CoA synthetase
HVO_1373	*acd4*	LIP	acyl-CoA dehydrogenase
HVO_1374	*acs4*	LIP	acyl-CoA synthetase
HVO_1387	*fadA1*	LIP	enoyl-CoA hydratase
HVO_1412	*mvaD, dmd*	LIP	phosphomevalonate decarboxylase
HVO_1444	*hbd3*	LIP	3-hydroxyacyl-CoA dehydrogenase
HVO_1447	*pccB1*	LIP	propionyl-CoA carboxylase carboxyltransferase component
HVO_1460	*fadA2*	LIP	enoyl-CoA hydratase
HVO_1585	*acs5*	LIP	acyl-CoA synthetase
HVO_1799		LIP	geranylgeranyl reductase / dolichol omega-reductase
HVO_1879	*fadA3*	LIP	enoyl-CoA hydratase
HVO_1914	*acaB3*	LIP	acetyl-CoA C-acyltransferase
HVO_1917	*acs6*	LIP	acyl-CoA synthetase
HVO_2315	*uppS1*	LIP	tritrans,polycis-undecaprenyl-diphosphate synthase
HVO_2318	*uppS2*	LIP	tritrans,polycis-undecaprenyl-diphosphate synthase
HVO_2419	*hmgB, mvaB*	LIP	hydroxymethylglutaryl-CoA synthase
HVO_2471	*pccB2*	LIP	propionyl-CoA carboxylase carboxyltransferase component
HVO_2506	*idiA*	LIP	isopentenyl-diphosphate delta-isomerase, type I
HVO_2524	*crtB*	LIP	phytoene synthase
HVO_2528	*crtI*	LIP	phytoene dehydrogenase (phytoene desaturase)
HVO_2583	*hmgA*	LIP	hydroxymethylglutaryl-CoA reductase (NADPH)
HVO_2716	*acd5*	LIP	acyl-CoA dehydrogenase
HVO_2725	*idsA1*	LIP	bifunctional short chain isoprenyl diphosphate synthase
HVO_2761	*mvk*	LIP	mevalonate kinase
HVO_2815	*hbd1*	LIP	3-hydroxyacyl-CoA dehydrogenase / enoyl-CoA hydratase
HVO_A0092	*acd6*	LIP	acyl-CoA dehydrogenase
HVO_A0097	*hbd2*	LIP	3-hydroxyacyl-CoA dehydrogenase / enoyl-CoA hydratase
HVO_A0156	*acs7*	LIP	acyl-CoA synthetase
HVO_A0158	*acs8*	LIP	acyl-CoA synthetase
HVO_A0505	*fadA4*	LIP	enoyl-CoA hydratase
HVO_A0522	*acaB4*	LIP	acetyl-CoA C-acyltransferase
HVO_A0524	*hbd4*	LIP	3-hydroxyacyl-CoA dehydrogenase
HVO_A0525	*fadA5*	LIP	enoyl-CoA hydratase
HVO_A0551	*acs9*	LIP	acyl-CoA synthetase
HVO_0011	*purO*	NUM	inosine-5'-monophosphate cyclohydrolase, archaeal-type
HVO_0334	*apt2*	NUM	purine phosphoribosyltransferase (adenine phosphoribosyltransferase, xanthine-guanine phosphoribosyltransferase)
HVO_0647	*guaAa1*	NUM	GMP synthase (glutamine-hydrolyzing) subunit A
HVO_0661	*dcd1*	NUM	dCTP deaminase (dUMP-forming)
HVO_0692	*mtaP*	NUM	5'-methylthioadenosine phosphorylase MtaP
HVO_0965	*deoA*	NUM	AMP phosphorylase (ribose-1,5-bisphosphate forming)
HVO_0976	*purK*	NUM	5-(carboxyamino)imidazole ribonucleotide synthase
HVO_0977	*purE*	NUM	N5-carboxyaminoimidazole ribonucleotide mutase
HVO_1048	*purL*	NUM	phosphoribosylformylglycinamidine synthase 2
HVO_1072	*apt1*	NUM	purine phosphoribosyltransferase (adenine phosphoribosyltransferase, xanthine-guanine phosphoribosyltransferase)
HVO_1084	*purB*	NUM	adenylosuccinate lyase
HVO_1085	*purNH*	NUM	phosphoribosylglycinamide formyltransferase / phosphoribosylaminoimidazolecarboxamide formyltransferase
HVO_1132	*purA*	NUM	adenylosuccinate synthase
HVO_1273	*guaB1*	NUM	inosine-5'-monophosphate dehydrogenase

HVO_1296		NUM	probable adenylate kinase
HVO_1454	*pyrB*	NUM	aspartate carbamoyltransferase catalytic subunit
HVO_1455	*pyrI*	NUM	aspartate carbamoyltransferase regulatory subunit
HVO_1557	*purM*	NUM	phosphoribosylformylglycinamidine cyclo-ligase
HVO_1579	*udp1*	NUM	uridine phosphorylase
HVO_1581	*cdd*	NUM	cytidine deaminase
HVO_1657	*purD*	NUM	phosphoribosylamine--glycine ligase
HVO_1816	*pyrC*	NUM	dihydroorotase
HVO_1866	*pyrH*	NUM	uridylate kinase
HVO_1893	*ham1*	NUM	non-canonical purine NTP pyrophosphatase
HVO_2188	*purQ*	NUM	phosphoribosylformylglycinamidine synthase 1
HVO_2189	*purS*	NUM	phosphoribosylformylglycinamidine synthase component PurS
HVO_2191	*purU*	NUM	formyltetrahydrofolate deformylase
HVO_2193	*purC*	NUM	phosphoribosylaminoimidazole-succinocarboxamide synthase
HVO_2250	*hpt*	NUM	purine phosphoribosyltransferase (adenine phosphoribosyltransferase, xanthine-guanine phosphoribosyltransferase)
HVO_2452	*nrdJ*	NUM	ribonucleoside-diphosphate reductase, adenosylcobalamin-dependent
HVO_2483	*guaD*	NUM	probable nucleoside deaminase (cytosine/guanine deaminase)
HVO_2494	*cmk*	NUM	cytidylate kinase
HVO_2496	*adk*	NUM	adenylate kinase
HVO_2577	*pyrF*	NUM	orotidine-5'-phosphate decarboxylase
HVO_2614	*udp2*	NUM	uridine phosphorylase
HVO_2624	*pyrG*	NUM	CTP synthase
HVO_2625	*guaAb*	NUM	GMP synthase (glutamine-hydrolyzing) subunit B
HVO_2721	*purF*	NUM	amidophosphoribosyltransferase
HVO_2740	*ndk*	NUM	nucleoside-diphosphate kinase
HVO_2797	*udk*	NUM	uridine kinase
HVO_2891	*tdk*	NUM	thymidine kinase
HVO_2918	*hts, thyA*	NUM	thymidylate synthase
HVO_2943	*pyrD*	NUM	dihydroorotate dehydrogenase (quinone)
HVO_2981	*upp*	NUM	uracil phosphoribosyltransferase
HVO_A0584	*guaB2*	NUM	inosine-5'-monophosphate dehydrogenase
Miscellaneous functions			
HVO_0019	*scm*	GEN	probable S-adenosylmethionine-dependent methyltransferase (homolog to 24-sterol C-methyltransferase)
HVO_0035		GEN	stomatin family protein
HVO_0055		GEN	CBS domain protein
HVO_0064		GEN	DUF296 family protein
HVO_0066		GEN	PQQ repeat protein
HVO_0069		GEN	sulfatase
HVO_0070		GEN	NifU C-terminal domain protein
HVO_0085		GEN	GlnK-type ammonia transport regulator
HVO_0096		GEN	arNOG08307 family NADH-binding domain protein
HVO_0099		GEN	helicase domain protein
HVO_0100		GEN	NMD3 family protein
HVO_0105		GEN	FAD-dependent oxidoreductase
HVO_0134		GEN	KH domain protein
HVO_0143	*phzF2*	GEN	PhzF family protein
HVO_0162		GEN	peptidase M48 family protein
HVO_0190		GEN	TIGR00268 family protein
HVO_0197	*purE2*	GEN	PurE family protein
HVO_0207		GEN	alpha/beta hydrolase fold protein
HVO_0208	*guaAa2*	GEN	glutamine amidotransferase (homolog to GMP synthase subunit A)
HVO_0212		GEN	homolog to lactoylglutathione lyase
HVO_0213	*dpsA2*	GEN	ferritin / Dps domain protein
HVO_0215		GEN	iron-sulfur protein (4Fe-4S)
HVO_0228		GEN	ParA domain protein
HVO_0237		GEN	UPF0761 family protein
HVO_0242	*pepB1*	GEN	aminopeptidase (homolog to leucyl aminopeptidase / aminopeptidase T)
HVO_0250		GEN	DUF21/CBS domain protein
HVO_0255		GEN	spermine/spermidine synthase family protein
HVO_0279		GEN	homolog to HGPV1-ORF14
HVO_0285		GEN	probable metalloprotease
HVO_0286		GEN	CinA N-terminal domain protein
HVO_0296		GEN	probable oxidoreductase (short-chain dehydrogenase family)
HVO_0302		GEN	DUF373 family protein
HVO_0322	*minD4*	GEN	MinD/ParA domain protein
HVO_0328	*galE1*	GEN	NAD-dependent epimerase/dehydratase
HVO_0330		GEN	DUF502 family protein
HVO_0357		GEN	ACT domain protein
HVO_0388	*vacB*	GEN	homolog to ribonuclease R
HVO_0390		GEN	PUA domain protein

HVO_0396		GEN	FAD-dependent oxidoreductase (homolog to geranylgeranyl reductase)
HVO_0397	*ygfZ*	GEN	homolog to aminomethyltransferase (homolog to glycine cleavage system protein T)
HVO_0401	*uspA2*	GEN	UspA domain protein
HVO_0402		GEN	DUF124 family protein
HVO_0405		GEN	NifU C-terminal domain protein
HVO_0408		GEN	PrsW family protein
HVO_0414		GEN	peptidase M24 family protein
HVO_0418		GEN	homolog to haloviral protein
HVO_0419		GEN	peptidase M20 family protein
HVO_0421		GEN	arNOG04375 family protein (homolog to PilT-type ATPase)
HVO_0425		GEN	homolog to catechol-2,3-dioxygenase
HVO_0431		GEN	HAD superfamily hydrolase
HVO_0441		GEN	probable metallo-beta-lactamase family hydrolase
HVO_0467	*pchB*	GEN	PhoU/TrkA-C domain protein
HVO_0469		GEN	AstE family protein
HVO_0477	*pepB3*	GEN	aminopeptidase (homolog to leucyl aminopeptidase / aminopeptidase T)
HVO_0483	*rimK2*	GEN	RimK family protein
HVO_0491		GEN	FMN-binding domain protein
HVO_0493		GEN	HTH domain protein
HVO_0511		GEN	NAD-dependent epimerase/dehydratase
HVO_0566		GEN	glycoside transferase domain protein
HVO_0585		GEN	probable oxidoreductase (aldo-keto reductase family protein)
HVO_0600		GEN	ACT domain protein
HVO_0611		GEN	DUF112 family protein
HVO_0635		GEN	DUF4129 domain protein
HVO_0648		GEN	DUF2070 family protein
HVO_0655		GEN	NAD-dependent epimerase/dehydratase
HVO_0668	*oadhB3*	GEN	2-oxoacid dehydrogenase E1 component beta subunit
HVO_0669	*oadhA3*	GEN	2-oxoacid dehydrogenase E1 component alpha subunit
HVO_0685		GEN	DUF3100 family protein
HVO_0687	*amiB*	GEN	M20 family amidohydrolase
HVO_0694	*gptA2*	GEN	probable phosphoribosyltransferase
HVO_0696	*phzF1*	GEN	PhzF family protein
HVO_0697		GEN	PAC2 family protein
HVO_0702		GEN	DUF2298 family protein
HVO_0704	*dgs*	GEN	probable glycosyltransferase, type 2
HVO_0721		GEN	PHP domain protein
HVO_0722		GEN	DUF457 family protein
HVO_0724	*arsA1*	GEN	ArsA family ATPase
HVO_0725		GEN	probable oxidoreductase (short-chain dehydrogenase family)
HVO_0727		GEN	rhomboid family protein
HVO_0736		GEN	DUF302 family protein
HVO_0740		GEN	DUF82 family protein
HVO_0743		GEN	probable phosphodiesterase
HVO_0744		GEN	homolog to carboxylate-amine ligase
HVO_0745	*ftsZ3*	GEN	FtsZ family protein, type III
HVO_0755		GEN	NAD-dependent epimerase/dehydratase
HVO_0756		GEN	phosphoesterase RecJ domain protein
HVO_0757		GEN	probable oxidoreductase (aldo-keto reductase family protein)
HVO_0760	*wcaA*	GEN	probable glycosyltransferase, type 2
HVO_0763		GEN	YphA family protein
HVO_0765	*soxB1*	GEN	FAD-dependent oxidoreductase
HVO_0768		GEN	radical SAM domain protein
HVO_0769		GEN	TRAM domain protein
HVO_0771		GEN	probable metallo-beta-lactamase family hydrolase
HVO_0773	*pnm*	GEN	probable S-adenosylmethionine-dependent methyltransferase
HVO_0774		GEN	probable glycosyltransferase, type 2
HVO_0781		GEN	S-adenosylmethionine hydroxide adenosyltransferase family protein
HVO_0801		GEN	stomatin family protein
HVO_0814		GEN	DnaJ N-terminal domain protein
HVO_0824	*rfbU*	GEN	probable glycosyltransferase, type 1
HVO_0826		GEN	peptidase M42 family protein
HVO_0829		GEN	peptidase S9 family protein
HVO_0832		GEN	alpha/beta hydrolase fold protein
HVO_0860	*sufB1*	GEN	SufB domain protein
HVO_0861	*sufB2, sufD*	GEN	SufB domain protein
HVO_0865		GEN	FkbM family methyltransferase
HVO_0866		GEN	homolog to alanine-tRNA ligase
HVO_0867		GEN	HD family hydrolase
HVO_0868		GEN	NAD-dependent epimerase/dehydratase

HVO_0874	*epf1*	GEN	beta-lactamase domain protein
HVO_0879		GEN	DUF371 family protein
HVO_0882		GEN	DUF373 family protein
HVO_0889		GEN	FAD-dependent oxidoreductase
HVO_0911	*drg*	GEN	GTP-binding protein Drg
HVO_0913		GEN	MATE efflux family protein
HVO_0914	*glo5*	GEN	probable lyase (homolog to lactoylglutathione lyase)
HVO_0931	*uspA6*	GEN	UspA domain protein
HVO_0937		GEN	FMN-binding domain protein
HVO_0960		GEN	probable oxidoreductase (aldo-keto reductase family protein)
HVO_0973	*aspC3*	GEN	pyridoxal phosphate-dependent aminotransferase (probable aspartate aminotransferase)
HVO_0975		GEN	homolog to membrane-bound mannosyltransferase
HVO_0990		GEN	DHH family phosphoesterase
HVO_0996		GEN	DUF21/CBS domain protein
HVO_0999		GEN	DRTGG domain protein
HVO_1007		GEN	homolog to quinoprotein glucose dehydrogenase
HVO_1008		GEN	GFO family oxidoreductase
HVO_1009	*aad1*	GEN	probable oxidoreductase (aldo-keto reductase family protein)
HVO_1018		GEN	RecJ domain protein
HVO_1020		GEN	HEAT-PBS family protein
HVO_1022		GEN	NamA family oxidoreductase (homolog to old yellow enzyme)
HVO_1038	*udg4*	GEN	uracil-DNA glycosylase superfamily protein
HVO_1040		GEN	DnaJ domain protein
HVO_1041		GEN	probable metallo-beta-lactamase family hydrolase
HVO_1049		GEN	PHP domain protein
HVO_1055	*trkA1*	GEN	TrkA domain protein
HVO_1058	*trkA2*	GEN	TrkA domain protein
HVO_1061	*trxB2*	GEN	oxidoreductase (homolog to thioredoxin-disulfide reductase)
HVO_1073	*trxB2*	GEN	ThiJ/PfpI family protein
HVO_1082	*pyrE1*	GEN	homolog to orotate phosphoribosyltransferase
HVO_1087	*uspA10*	GEN	UspA domain protein
HVO_1093		GEN	probable S-adenosylmethionine-dependent methyltransferase
HVO_1113	*ftsZ5*	GEN	FtsZ family protein, type III
HVO_1137	*pchA1*	GEN	ion channel pore / TrkA domain protein
HVO_1164		GEN	HD family hydrolase
HVO_1168		GEN	probable glycosyltransferase, type 1
HVO_1169	*sqdX*	GEN	probable glycosyltransferase, type 1
HVO_1172	*galE2*	GEN	NAD-dependent epimerase/dehydratase
HVO_1175		GEN	DUF2110 family protein
HVO_1177		GEN	CinA N-terminal domain protein
HVO_1179		GEN	homolog to NAD kinase
HVO_1184		GEN	DUF1511 family protein
HVO_1198	*uspA11*	GEN	UspA domain protein
HVO_1209		GEN	HTH domain protein
HVO_1228	*hcpE*	GEN	halocyanin
HVO_1238		GEN	probable oxidoreductase (aldo-keto reductase family protein)
HVO_1241	*prrC*	GEN	SCO1/SenC/PrrC family protein
HVO_1250		GEN	redoxin domain protein
HVO_1260		GEN	von Willebrand factor type A domain protein
HVO_1266	*aspC2*	GEN	pyridoxal phosphate-dependent aminotransferase (probable aspartate aminotransferase)
HVO_1281		GEN	probable glycosyltransferase, type 1
HVO_1283		GEN	oxidoreductase (homolog to zinc-containing alcohol dehydrogenase / threonine 3-dehydrogenase)
HVO_1289		GEN	OsmC domain protein
HVO_1291	*hpcE*	GEN	fumarylacetoacetase family protein
HVO_1294		GEN	GMC family oxidoreductase
HVO_1309	*pepQ*	GEN	peptidase M24 family protein
HVO_1311		GEN	probable oxidoreductase (short-chain dehydrogenase family)
HVO_1313		GEN	probable S-adenosylmethionine-dependent methyltransferase
HVO_1327	*cdc48c*	GEN	AAA-type ATPase (CDC48 subfamily)
HVO_1340		GEN	probable glycosyltransferase, type 1
HVO_1344		GEN	SBDS family protein
HVO_1346	*hflX*	GEN	GTP-binding protein HflX
HVO_1368		GEN	probable metallo-beta-lactamase family hydrolase
HVO_1369		GEN	DUF124 family protein
HVO_1377		GEN	UPF0145 family protein
HVO_1381	*mdmC*	GEN	probable methyltransferase
HVO_1391	*apa*	GEN	NUDIX family hydrolase
HVO_1395		GEN	M20 family amidohydrolase (homolog to indole-3-acetyl-aspartic acid hydrolase)
HVO_1396		GEN	FAD-dependent oxidoreductase (homolog to geranylgeranyl reductase)
HVO_1407		GEN	HTH domain protein

HVO_1459		GEN	ThuA family protein
HVO_1463	*trxB4*	GEN	oxidoreductase (homolog to thioredoxin-disulfide reductase)
HVO_1477	*comA*	GEN	endonuclease domain protein
HVO_1479		GEN	CHP03663 family protein
HVO_1481	*uspA13*	GEN	UspA domain protein
HVO_1483		GEN	metallo-beta-lactamase superfamily domain protein (nonfunctional)
HVO_1491		GEN	HAD superfamily hydrolase
HVO_1532		GEN	DUF368 family protein
HVO_1534		GEN	probable S-adenosylmethionine-dependent methyltransferase
HVO_1554	*traB*	GEN	TraB family protein
HVO_1559		GEN	HTH/CBS domain protein
HVO_1568		GEN	alpha/beta hydrolase fold protein
HVO_1574		GEN	NUDIX family hydrolase
HVO_1576	*galE3*	GEN	NAD-dependent epimerase/dehydratase
HVO_1584		GEN	GNAT family acetyltransferase
HVO_1593	*ppd*	GEN	PAC2 family protein
HVO_1596		GEN	DUF790 family protein
HVO_1604	*aspC1*	GEN	pyridoxal phosphate-dependent aminotransferase (probable aspartate aminotransferase)
HVO_1626		GEN	homolog to UPF0173 family metal-dependent hydrolase
HVO_1656		GEN	YyaL family protein
HVO_1658	*yvoF*	GEN	O-acetyltransferase (homolog to galactoside O-acetyltransferase)
HVO_1660		GEN	DisA-N domain protein
HVO_1690		GEN	DHH family phosphoesterase
HVO_1692		GEN	probable iron-sulfur protein (4Fe-4S)
HVO_1697		GEN	FAD-dependent oxidoreductase (GlcD/DLD_GlcF/GlpC domain fusion protein)
HVO_1698		GEN	CBS/parB domain protein
HVO_1699		GEN	pyridoxal phosphate-dependent aminotransferase (homolog to histidinol-phosphate aminotransferase / aspartate aminotransferase)
HVO_1704		GEN	homolog to arabinopyranose mutase
HVO_1726		GEN	HTH domain protein
HVO_1746		GEN	DUF2800 family protein
HVO_1758	*trxB5*	GEN	oxidoreductase (homolog to thioredoxin-disulfide reductase)
HVO_1803		GEN	conserved hypothetical protein
HVO_1809	*entB2*	GEN	isochorismatase family protein
HVO_1821	*pat2*	GEN	GNAT family acetyltransferase Pat2
HVO_1822	*uspA15*	GEN	UspA domain protein
HVO_1823	*uspA16*	GEN	UspA domain protein
HVO_1829	*pepB2*	GEN	aminopeptidase (homolog to leucyl aminopeptidase / aminopeptidase T)
HVO_1838		GEN	DUF262/DUF1524 domain protein (nonfunctional)
HVO_1849	*ywaD2*	GEN	probable M28 family peptidase (homolog to aminopeptidase YwaD)
HVO_1853	*uspA17*	GEN	UspA domain protein
HVO_1859		GEN	UPF0104 family protein
HVO_1862		GEN	probable metalloprotease
HVO_1870		GEN	probable metalloprotease
HVO_1874		GEN	probable oxidoreductase (aldo-keto reductase family protein)
HVO_1875		GEN	O-acetyltransferase (homolog to galactoside O-acetyltransferase)
HVO_1885	*trkA3*	GEN	TrkA domain protein
HVO_1907	*cdc48d*	GEN	arNOG05511 family protein (AAA-type ATPase core domain protein)
HVO_1918		GEN	probable metallo-beta-lactamase family hydrolase
HVO_1925	*gbp3*	GEN	probable GTP-binding protein
HVO_1926		GEN	SIMPL domain protein
HVO_1927		GEN	DUF389 family protein
HVO_1943		GEN	endoisopeptidase/DUF4129 domain protein
HVO_1948		GEN	NUDIX family hydrolase
HVO_1966		GEN	Abi/CAAX family protein
HVO_1972		GEN	GNAT family acetyltransferase
HVO_2013	*ftsZ7*	GEN	FtsZ family protein, type III
HVO_2020		GEN	DUF502 family protein
HVO_2027		GEN	YphA family protein
HVO_2040	*galE4*	GEN	NAD-dependent epimerase/dehydratase
HVO_2068	*ftsZ8*	GEN	FtsZ family protein, type III
HVO_2081		GEN	pectin lyase domain protein
HVO_2088	*xloA*	GEN	glycoside hydrolase family protein
HVO_2091	*gabT2*	GEN	pyridoxal phosphate-dependent aminotransferase
HVO_2095		GEN	probable oxidoreductase (zinc-containing alcohol dehydrogenase family)
HVO_2096		GEN	NAD-dependent epimerase/dehydratase
HVO_2127		GEN	M20 family amidohydrolase (homolog to indole-3-acetyl-aspartic acid hydrolase)
HVO_2147	*norB*	GEN	nitric oxide reductase (cytochrome c)
HVO_2153	*mcoA*	GEN	probable copper-containing oxidoreductase
HVO_2156	*uspA18*	GEN	UspA domain protein

HVO_2171		GEN	DUF58 family protein
HVO_2173		GEN	DUF1616 family protein
HVO_2187		GEN	CHP01210 family protein
HVO_2204	*ftsZ4*	GEN	FtsZ family protein, type III
HVO_2205	*nolA*	GEN	arNOG06768 family NADH-binding domain protein
HVO_2211	*trkA4*	GEN	TrkA domain protein
HVO_2225		GEN	AstE family protein
HVO_2226	*trpD2*	GEN	probable phosphoribosyltransferase (homolog to anthranilate phosphoribosyltransferase)
HVO_2228		GEN	PHP domain protein
HVO_2229		GEN	flavin-containing amine-oxidoreductase
HVO_2239	*uspA19*	GEN	UspA domain protein
HVO_2244	*mhpD*	GEN	fumarylacetoacetase family protein
HVO_2256		GEN	DUF262 family protein
HVO_2275		GEN	N-6 adenine-specific DNA methylase domain protein
HVO_2281		GEN	homolog to transfer complex protein
HVO_2307	*pgp*	GEN	HAD superfamily hydrolase
HVO_2316		GEN	serpin family protein
HVO_2322		GEN	YfiH family protein
HVO_2323		GEN	DUF3311 family protein
HVO_2328	*entB1*	GEN	isochorismatase family protein
HVO_2340		GEN	amine oxidase family protein
HVO_2345	*noxA*	GEN	flavin-dependent pyridine nucleotide oxidoreductase
HVO_2350		GEN	YyaL family protein
HVO_2351	*fxsA*	GEN	FxsA domain protein
HVO_2356		GEN	GNAT family acetyltransferase
HVO_2360		GEN	peptidase S66 family protein
HVO_2367		GEN	arNOG05179 family protein (DUF87-related AAA-type ATPase)
HVO_2370		GEN	HAD superfamily hydrolase
HVO_2380	*cdc48a*	GEN	AAA-type ATPase (CDC48 subfamily)
HVO_2381		GEN	UPF0272 family protein
HVO_2384		GEN	CBS domain protein
HVO_2410		GEN	UPF0753 family protein
HVO_2425		GEN	GNAT family acetyltransferase
HVO_2435		GEN	peptidase M24 family protein
HVO_2448	*aad2*	GEN	probable oxidoreductase (aldo-keto reductase family protein)
HVO_2461	*gul*	GEN	CBS domain protein
HVO_2468		GEN	probable oxidoreductase (short-chain dehydrogenase family)
HVO_2481		GEN	HD family hydrolase
HVO_2482		GEN	HD family hydrolase
HVO_2495		GEN	DUF106 family protein
HVO_2501		GEN	alpha/beta hydrolase fold protein
HVO_2504	*atsC*	GEN	probable oxidoreductase (short-chain dehydrogenase family)
HVO_2510		GEN	GNAT family acetyltransferase
HVO_2521		GEN	DUF21/CBS domain protein
HVO_2529		GEN	probable oxidoreductase (short-chain dehydrogenase family)
HVO_2530		GEN	homolog to phosphoribosylamine--glycine ligase
HVO_2534	*uspA24*	GEN	UspA domain protein
HVO_2536		GEN	CHP00300 family protein
HVO_2538		GEN	alpha/beta hydrolase fold protein
HVO_2571		GEN	UPF0761 family protein
HVO_2575		GEN	homolog to translation elongation factor aEF-1 alpha subunit
HVO_2590		GEN	probable oxidoreductase (short-chain dehydrogenase family)
HVO_2606		GEN	PQQ repeat protein
HVO_2607		GEN	PQQ repeat protein
HVO_2616	*pchA2*	GEN	ion channel pore / TrkA domain protein
HVO_2617		GEN	TrkA-C domain protein
HVO_2618		GEN	TrkA-C domain protein
HVO_2622	*ara*	GEN	probable oxidoreductase (aldo-keto reductase family protein)
HVO_2628		GEN	GHMP family kinase (homolog to beta-ribofuranosylaminobenzene 5'-phosphate synthase)
HVO_2636	*pspA*	GEN	PspA domain protein
HVO_2638	*tcbD*	GEN	homolog to mandelate racemase / homolog to muconate lactonizing enzyme
HVO_2639		GEN	DUF1611 family protein
HVO_2650		GEN	FAD-dependent oxidoreductase (homolog to geranylgeranyl reductase)
HVO_2654	*narC2*	GEN	homolog to respiratory nitrate reductase b-type cytochrome subunit
HVO_2655	*narB2*	GEN	Rieske-type [2Fe-2S] iron-sulfur protein
HVO_2661		GEN	aminotransferase class V (serine--pyruvate aminotransferase / alanine--glyoxylate aminotransferase)
HVO_2662	*glo1*	GEN	homolog to lactoylglutathione lyase
HVO_2663		GEN	probable oxidoreductase (aldo-keto reductase family protein)
HVO_2665	*hpcH*	GEN	HpcH/HpaI aldolase family protein
HVO_2670		GEN	FAD-dependent oxidoreductase (GlcD/DLD_GlcF/GlpC domain fusion protein)

HVO_2671		GEN	aminotransferase class V (serine--pyruvate aminotransferase / alanine--glyoxylate aminotransferase)
HVO_2681		GEN	HTH domain protein
HVO_2683		GEN	5'-nucleotidase family hydrolase
HVO_2690		GEN	GFO family oxidoreductase
HVO_2699	*bcp1*	GEN	homolog to peroxiredoxin
HVO_2700	*cdc48b*	GEN	AAA-type ATPase (CDC48 subfamily)
HVO_2702		GEN	alpha/beta hydrolase fold protein
HVO_2707		GEN	GFO family oxidoreductase
HVO_2708		GEN	YfiH family protein
HVO_2711		GEN	DoxX domain protein
HVO_2724		GEN	beta-lactamase domain protein
HVO_2727		GEN	cyclase family protein
HVO_2732		GEN	FixC family protein
HVO_2759		GEN	peptidase M42 family protein
HVO_2763		GEN	homolog to ribonuclease Z
HVO_2772		GEN	rhodanese domain protein / probable metallo-beta-lactamase family hydrolase
HVO_2812		GEN	AstE family protein
HVO_2813		GEN	RimK family protein
HVO_2846	*spoVR*	GEN	SpoVR family protein
HVO_2852		GEN	succinylglutamate desuccinylase/aspartoacylase family protein
HVO_2859		GEN	DUF63 family protein
HVO_2871	*gabT3*	GEN	pyridoxal phosphate-dependent aminotransferase
HVO_2875		GEN	probable S-adenosylmethionine-dependent methyltransferase
HVO_2876		GEN	DUF3054 family protein
HVO_2881		GEN	DUF4013 family protein
HVO_2882		GEN	DUF4013 family protein
HVO_2886		GEN	GNAT family acetyltransferase
HVO_2888	*elp3*	GEN	homolog to elongator complex protein ELP3
HVO_2889		GEN	RecJ domain protein
HVO_2905	*maoC*	GEN	MaoC family protein
HVO_2912		GEN	ThiJ/PfpI domain protein
HVO_2916		GEN	probable oxidoreductase (short-chain dehydrogenase family)
HVO_2932	*gbp4*	GEN	GTP-binding protein
HVO_2977	*arsA2*	GEN	ArsA family ATPase
HVO_2979		GEN	CobW domain protein
HVO_3008		GEN	UPF0148 family protein
HVO_A0003	*parA3*	GEN	ParA domain protein
HVO_A0009		GEN	probable S-adenosylmethionine-dependent methyltransferase
HVO_A0035	*ftsZ6*	GEN	FtsZ family protein, type III
HVO_A0047	*uspA26*	GEN	UspA domain protein
HVO_A0049		GEN	DUF21/CBS domain protein
HVO_A0087	*xlnF*	GEN	fumarylacetoacetase family protein
HVO_A0088		GEN	FAD-dependent oxidoreductase (GlcD/DLD_GlcF/GlpC domain fusion protein)
HVO_A0119		GEN	cyclase family protein
HVO_A0153	*uspA28*	GEN	UspA domain protein
HVO_A0155		GEN	DUF4212 family protein
HVO_A0159	*aspC4*	GEN	pyridoxal phosphate-dependent aminotransferase (probable aspartate aminotransferase)
HVO_A0162	*matE1*	GEN	MATE efflux family protein
HVO_A0169		GEN	DUF106 family protein
HVO_A0218		GEN	GFO family oxidoreductase
HVO_A0254		GEN	DUF234 domain protein
HVO_A0268	*fuca*	GEN	class II aldolase (homolog to L-fuculose-phosphate aldolase)
HVO_A0273		GEN	ThuA family protein
HVO_A0286		GEN	DUF187 family protein
HVO_A0287		GEN	homolog to mandelate racemase / homolog to muconate lactonizing enzyme
HVO_A0288		GEN	probable oxidoreductase (short-chain dehydrogenase family)
HVO_A0295	*amaB2*	GEN	amidase (hydantoinase/carbamoylase family)
HVO_A0295_A		GEN	luciferase family protein
HVO_A0296		GEN	probable oxidoreductase (short-chain dehydrogenase family)
HVO_A0301		GEN	polysaccharide deacetylase family protein
HVO_A0302	*ansB*	GEN	asparaginase/glutaminase family protein
HVO_A0304	*groL*	GEN	homolog to thermosome
HVO_A0362		GEN	PQQ repeat protein
HVO_A0401		GEN	Fido domain protein
HVO_A0415		GEN	cyclase family protein
HVO_A0434		GEN	probable S-adenosylmethionine-dependent methyltransferase (nonfunctional)
HVO_A0439		GEN	DUF2800 family protein
HVO_A0440		GEN	DUF790 family protein
HVO_A0452	*uspA30*	GEN	UspA domain protein
HVO_A0458	*sojE*	GEN	ParA domain protein

HVO_A0472	*trxB6*	GEN	oxidoreductase (homolog to thioredoxin-disulfide reductase)
HVO_A0485		GEN	aminotransferase class V
HVO_A0489	*nifV*	GEN	homolog to homocitrate synthase
HVO_A0496	*uspA31*	GEN	UspA domain protein
HVO_A0517		GEN	luciferase-type oxidoreductase
HVO_A0523		GEN	UPF0219 family protein
HVO_A0535		GEN	peptidase M24 family protein
HVO_A0547		GEN	DUF1028 family protein
HVO_A0548	*doxD1*	GEN	YphA family protein
HVO_A0555		GEN	DUF162 family protein
HVO_A0569	*baiF1*	GEN	family 3 CoA transferase
HVO_A0570		GEN	probable metal-dependent hydrolase
HVO_A0571		GEN	isochorismatase family protein
HVO_A0591		GEN	DICT domain protein
HVO_A0601	*nosL2*	GEN	NosL family protein
HVO_A0618		GEN	flavin-dependent pyridine nucleotide oxidoreductase (homolog to coenzyme A disulfide reductase)
HVO_A0636	*ybaK*	GEN	YbaK domain protein
HVO_B0018	*parA2*	GEN	ParA domain protein
HVO_B0025		GEN	oxidoreductase (homolog to zinc-containing alcohol dehydrogenase)
HVO_B0026		GEN	probable oxidoreductase (short-chain dehydrogenase family)
HVO_B0031		GEN	probable oxidoreductase (short-chain dehydrogenase family)
HVO_B0052		GEN	PQQ repeat protein
HVO_B0070	*gabT1*	GEN	pyridoxal phosphate-dependent aminotransferase
HVO_B0071	*adh2*	GEN	oxidoreductase (homolog to zinc-containing alcohol dehydrogenase)
HVO_B0085		GEN	glycoside hydrolase family protein
HVO_B0087		GEN	homolog to mandelate racemase / homolog to muconate lactonizing enzyme
HVO_B0096		GEN	homolog to sugar epimerase/dehydratase
HVO_B0102		GEN	glycosidase family protein
HVO_B0110		GEN	oxidoreductase (homolog to zinc-containing alcohol dehydrogenase)
HVO_B0111		GEN	homolog to mandelate racemase / homolog to muconate lactonizing enzyme
HVO_B0115		GEN	fumarylacetoacetase family protein
HVO_B0116		GEN	probable oxidoreductase (short-chain dehydrogenase family)
HVO_B0127		GEN	oxidoreductase (luciferase family protein)
HVO_B0130		GEN	DUF296 family protein
HVO_B0132		GEN	PQQ repeat protein
HVO_B0138		GEN	PQQ repeat protein
HVO_B0139		GEN	PQQ repeat protein
HVO_B0146		GEN	aminotransferase class V
HVO_B0153	*capB*	GEN	CapB domain protein
HVO_B0179		GEN	YfiH family protein
HVO_B0183	*racD*	GEN	YgeA family protein
HVO_B0202		GEN	sensor box protein
HVO_B0203		GEN	FAD-dependent oxidoreductase (GlcD/DLD_GlcF/GlpC domain fusion protein)
HVO_B0216		GEN	UPF0261 family protein
HVO_B0243	*cobW*	GEN	CobW domain protein
HVO_B0248		GEN	probable oxidoreductase (short-chain dehydrogenase family)
HVO_B0263		GEN	M20 family amidohydrolase (homolog to indole-3-acetyl-aspartic acid hydrolase)
HVO_B0265		GEN	FAD-dependent oxidoreductase (GlcD/DLD_GlcF/GlpC domain fusion protein)
HVO_B0267		GEN	peptidase M24 family protein
HVO_B0277		GEN	HTH domain protein
HVO_B0321		GEN	GFO family oxidoreductase
HVO_B0325		GEN	glycoside hydrolase family protein
HVO_B0338		GEN	probable hydro-lyase (homolog to altronate dehydratase)
HVO_B0345		GEN	homolog to mandelate racemase / homolog to muconate lactonizing enzyme
HVO_B0359		GEN	YidE family protein
HVO_B0375		GEN	Spo0M family protein
HVO_B0376		GEN	probable oxidoreductase (aldo-keto reductase family protein)
HVO_C0042		GEN	helicase domain protein
HVO_C0044		GEN	PLD domain protein
HVO_C0060		GEN	CopG family protein
HVO_C0067	*soxA3*	GEN	FAD-dependent oxidoreductase
HVO_C0069	*gfo1*	GEN	GFO family oxidoreductase
HVO_0275		ISH	ISH5-type transposase ISHvo11
HVO_1150		ISH	IS200-type transposase
HVO_2051		ISH	ISH5-type transposase ISHvo11
HVO_2075		ISH	IS1341-type transposase
HVO_2090_A		ISH	IS1341-type transposase (nonfunctional)
HVO_A0018		ISH	IS1341-type transposase
HVO_A0238		ISH	ISH5-type transposase ISHvo11
HVO_A0260		ISH	ISH5-type transposase ISHvo11

HVO_B0241		ISH	IS1341-type transposase
HVO_C0020_A		ISH	ISH14-type transposase HfIRS8 (nonfunctional)
HVO_C0053		ISH	ISH5-type transposase ISHvo11
HVO_0001	*orc1*	MIS	Orc1-type DNA replication protein
HVO_0024	*tssA2*	MIS	thiosulfate sulfurtransferase
HVO_0025	*tssA1*	MIS	thiosulfate sulfurtransferase
HVO_0034	*act1*	MIS	acyl-CoA thioester hydrolase
HVO_0038	*cyc1*	MIS	cytochrome P450
HVO_0102	*htpX1*	MIS	HtpX-like protease
HVO_0135	*rio1, pkn2*	MIS	RIO-type serine/threonine protein kinase
HVO_0165	*mtaD*	MIS	5-methylthioadenosine/S-adenosylhomocysteine deaminase
HVO_0177	*arsC*	MIS	arsenate reductase (glutaredoxin)
HVO_0194	*orc9*	MIS	Orc1-type DNA replication protein
HVO_0283	*ashA*	MIS	archaea-specific helicase AshA
HVO_0406	*aubA*	MIS	RNA-binding protein AU-1
HVO_0417	*cxp*	MIS	metal-dependent carboxypeptidase
HVO_0429	*amzA*	MIS	archaemetzincin
HVO_0433	*npdG*	MIS	F420H2:NADP oxidoreductase
HVO_0459	*yjgF1*	MIS	enamine/imine deaminase
HVO_0486	*nucS*	MIS	endonuclease NucS
HVO_0536	*dpsA1*	MIS	ferritin
HVO_0569	*rio2, pkn1*	MIS	RIO-type serine/threonine protein kinase
HVO_0572	*lpl*	MIS	lipoate-protein ligase
HVO_0581	*ftsZ2*	MIS	cell division protein FtsZ, type II
HVO_0620	*pilB1*	MIS	type IV pilus biogenesis complex ATPase subunit
HVO_0634	*orc2*	MIS	Orc1-type DNA replication protein
HVO_0639	*moxR1*	MIS	AAA-type ATPase (MoxR subfamily)
HVO_0670		MIS	glutathione S-transferase
HVO_0682	*mrr*	MIS	probable Mrr family endonuclease
HVO_0716	*dtdA*	MIS	D-tyrosyl-tRNA(Tyr) deacylase
HVO_0729	*ipp, ppa*	MIS	inorganic pyrophosphatase
HVO_0748	*pilB2*	MIS	type IV pilus biogenesis complex ATPase subunit
HVO_0798	*aglD*	MIS	dolichyl-phosphate mannosyltransferase AglD
HVO_0836	*ywaD1*	MIS	probable M28 family peptidase (homolog to aminopeptidase YwaD)
HVO_0859	*sufC*	MIS	FeS assembly ATPase SufC
HVO_0876	*mgsA*	MIS	methylglyoxal synthase
HVO_0881	*sppA1*	MIS	signal peptide peptidase SppA
HVO_0916	*dph5*	MIS	diphthine synthase
HVO_0935	*fdhA*	MIS	formate dehydrogenase alpha subunit
HVO_0952	*cynT*	MIS	carbonic anhydrase
HVO_0972	*pilA1*	MIS	pilin PilA
HVO_0997		MIS	peptidase M24 family protein
HVO_1031	*trxB1*	MIS	thioredoxin-disulfide reductase
HVO_1033	*pilC3*	MIS	type IV pilus biogenesis complex membrane subunit
HVO_1034	*pilB3*	MIS	type IV pilus biogenesis complex ATPase subunit
HVO_1047	*qor2*	MIS	NADPH:quinone reductase
HVO_1077	*dph6*	MIS	diphthamide biosynthesis protein Dph6
HVO_1123	*trxB3*	MIS	thioredoxin-disulfide reductase
HVO_1160	*pilB4*	MIS	type IV pilus biogenesis complex ATPase subunit
HVO_1189	*aldH2*	MIS	aldehyde dehydrogenase
HVO_1212	*cirA*	MIS	KaiC-type circadian regulator CirA
HVO_1245		MIS	probable disulfide bond formation protein
HVO_1257	*moxR2*	MIS	AAA-type ATPase (MoxR subfamily)
HVO_1287	*pfpI*	MIS	PfpI family protease
HVO_1314	*surE1*	MIS	5'-nucleotidase SurE
HVO_1333	*lhr2*	MIS	ATP-dependent DNA helicase
HVO_1517	*aglJ*	MIS	dolichyl-phosphate hexosyltransferase AglJ
HVO_1522	*aglP*	MIS	hexuronic acid methyltransferase AglP
HVO_1523	*aglQ*	MIS	agl cluster protein AglQ
HVO_1523_A	*aglE*	MIS	glycosyltransferase AglE
HVO_1528	*aglI*	MIS	glycosyltransferase AglI
HVO_1529	*aglG*	MIS	glycosyltransferase AglG
HVO_1530	*aglB*	MIS	dolichyl-monophosphooligosaccharide--protein glycotransferase AglB
HVO_1537	*orc15*	MIS	Orc1-type DNA replication protein
HVO_1544	*dhaM*	MIS	probable phosphoenolpyruvate-protein phosphoryltransferase
HVO_1550	*act2*	MIS	acyl-CoA thioester hydrolase
HVO_1558	*cyc2*	MIS	cytochrome P450
HVO_1613	*gst*	MIS	probable glycosyltransferase, type 2
HVO_1631	*dph2*	MIS	S-adenosyl-L-methionine:L-histidine 3-amino-3-carboxypropyltransferase
HVO_1637	*mtfK2*	MIS	FKBP-type peptidylprolyl isomerase

HVO_1648	*cyaB*	MIS	adenylate cyclase
HVO_1649	*mat*	MIS	methionine adenosyltransferase
HVO_1650	*ppk1*	MIS	polyphosphate kinase
HVO_1668	*gshA*	MIS	gamma-glutamylcysteine synthetase
HVO_1681	*ogg*	MIS	DNA N-glycosylase
HVO_1725	*orc5*	MIS	Orc1-type DNA replication protein
HVO_1778	*katG*	MIS	catalase-peroxidase
HVO_1788	*nirA1*	MIS	probable sulfite/nitrite reductase (ferredoxin)
HVO_1847	*pepF*	MIS	oligoendopeptidase PepF
HVO_1871	*pitA*	MIS	UPF0447 family protein
HVO_1908	*nasA*	MIS	assimilatory nitrate reductase
HVO_1911	*nasD, nirA2*	MIS	nitrite reductase (ferredoxin) (nonfunctional)
HVO_1932	*ddh*	MIS	2-D-hydroxyacid dehydrogenase
HVO_1937	*mer*	MIS	probable 5,10-methylenetetrahydrofolate reductase
HVO_1941		MIS	AAA-type ATPase (MoxR subfamily)
HVO_1987	*sppA2*	MIS	signal peptide peptidase SppA
HVO_2042	*orc4*	MIS	Orc1-type DNA replication protein
HVO_2046		MIS	sulfatase domain protein
HVO_2048		MIS	probable glycosyltransferase, type 1
HVO_2049	*rfbF*	MIS	probable glycosyltransferase, type 2
HVO_2053		MIS	probable glycosyltransferase, type 1
HVO_2060		MIS	NUDIX family hydrolase
HVO_2061	*dpm*	MIS	probable glycosyltransferase, type 2
HVO_2062	*pilA2*	MIS	pilin PilA
HVO_2141	*nirK*	MIS	nitrite reductase, copper-containing
HVO_2168	*moxR3*	MIS	AAA-type ATPase (MoxR subfamily)
HVO_2183	*aor4*	MIS	aldehyde ferredoxin oxidoreductase
HVO_2222	*ppiA*	MIS	CYPL-type peptidylprolyl isomerase
HVO_2269	*rmeR*	MIS	type I site-specific deoxyribonuclease subunit RmeR
HVO_2270	*rmeM*	MIS	type I restriction-modification system DNA-methyltransferase RmeM
HVO_2271	*rmeS*	MIS	type I site-specific deoxyribonuclease subunit RmeS
HVO_2297	*dhs1*	MIS	deoxyhypusine synthase
HVO_2385	*pilB5*	MIS	type IV pilus biogenesis complex ATPase subunit
HVO_2416	*tatD*	MIS	TatD-related deoxyribonuclease
HVO_2428	*adh1*	MIS	oxidoreductase (homolog to zinc-containing alcohol dehydrogenase)
HVO_2467	*qor3*	MIS	NADPH:quinone reductase
HVO_2485	*birA*	MIS	biotin--[acetyl-CoA-carboxylase] ligase
HVO_2502	*hypE2, hmf*	MIS	hydrogenase expression/formation protein
HVO_2598	*ppk2*	MIS	polyphosphate kinase
HVO_2600	*map*	MIS	methionyl aminopeptidase
HVO_2643	*qor1*	MIS	NADPH:quinone reductase
HVO_2682		MIS	dodecin
HVO_2723	*lsm, snp*	MIS	RNA-binding protein Lsm
HVO_2766	*lhr1*	MIS	ATP-dependent DNA helicase
HVO_2790	*mrp1*	MIS	ATP-binding protein Mrp
HVO_2848	*prkA1*	MIS	probable PrkA-type serine/threonine protein kinase
HVO_2849	*prkA2*	MIS	probable PrkA-type serine/threonine protein kinase
HVO_2904	*htpX2*	MIS	HtpX-like protease
HVO_2913	*sod2*	MIS	superoxide dismutase (Mn)
HVO_2929	*ssnA*	MIS	probable nucleoside deaminase (cytosine/guanine deaminase)
HVO_2941	*mc1B*	MIS	nonhistone chromosomal protein
HVO_2955		MIS	DEAD/DEAH box helicase
HVO_2960	*dsa1*	MIS	dihydrolipoamide S-acyltransferase
HVO_2993	*pibD, flaK*	MIS	prepilin/preflagellin peptidase
HVO_3010	*hef*	MIS	ATP-dependent RNA helicase/nuclease Hef
HVO_3012	*oapC*	MIS	origin-associated protein OapC
HVO_3013	*oapB*	MIS	origin-associated protein OapB
HVO_3014	*oapA*	MIS	origin-associated GTP-binding protein OapA
HVO_A0001	*orc3*	MIS	Orc1-type DNA replication protein
HVO_A0045	*htpX3*	MIS	HtpX-like protease
HVO_A0064	*orc13*	MIS	Orc1-type DNA replication protein
HVO_A0079		MIS	probable restriction/modification enzyme
HVO_A0205	*cas6*	MIS	CRISPR-associated endoribonuclease Cas6
HVO_A0206	*cas8b*	MIS	CRISPR-associated protein Cas8b
HVO_A0207	*cas7*	MIS	CRISPR-associated protein Cas7
HVO_A0208	*cas5*	MIS	CRISPR-associated endoribonuclease Cas5, Hmari subtype
HVO_A0209	*cas3*	MIS	CRISPR-associated helicase Cas3
HVO_A0228	*surE2*	MIS	5'-nucleotidase SurE
HVO_A0230	*msrA1*	MIS	peptide methionine sulfoxide reductase MsrA (S-form specific)
HVO_A0257	*orc7*	MIS	Orc1-type DNA replication protein

HVO_A0303	*pucH2*	MIS	probable allantoinase
HVO_A0326	*bgaH*	MIS	beta-D-galactosidase
HVO_A0475	*sod1*	MIS	superoxide dismutase (Mn)
HVO_A0486	*hypE1*	MIS	hydrogenase expression/formation protein
HVO_A0521	*paaK1*	MIS	phenylacetyl-coenzyme A ligase
HVO_A0536	*hyuB1*	MIS	N-methylhydantoinase (ATP-hydrolyzing) B
HVO_A0537	*hyuA1*	MIS	N-methylhydantoinase (ATP-hydrolyzing) A
HVO_A0633	*pilA6*	MIS	pilin PilA
HVO_B0001	*orc6*	MIS	Orc1-type DNA replication protein
HVO_B0044	*iucA*	MIS	siderophore biosynthesis protein IucA
HVO_B0045	*bdb*	MIS	diaminobutyrate decarboxylase
HVO_B0069		MIS	aldehyde dehydrogenase
HVO_B0076	*hyuA4*	MIS	N-methylhydantoinase (ATP-hydrolyzing) A
HVO_B0100	*aldH3, aldY3*	MIS	aldehyde dehydrogenase
HVO_B0149	*ohyA*	MIS	oleate hydratase
HVO_B0182	*dhs2*	MIS	deoxyhypusine synthase
HVO_B0205	*lccA*	MIS	laccase
HVO_B0256	*aor3*	MIS	aldehyde ferredoxin oxidoreductase
HVO_B0300	*pucL1*	MIS	uricase
HVO_B0371	*aldH1*	MIS	aldehyde dehydrogenase
HVO_C0001	*orc10*	MIS	Orc1-type DNA replication protein
HVO_C0040		MIS	site-specific DNA-methyltransferase (cytosine-specific)
HVO_C0057	*orc8*	MIS	Orc1-type DNA replication protein
Transport and cellular processes			
HVO_0915	*artA, cyo*	CE	archaeosortase A
HVO_2072	*csg*	CE	S-layer glycoprotein
HVO_0522	*hda1, hdal*	CP	HdaI-type histone deacetylase
HVO_0558	*ubaA, moeB*	CP	SAMP-activating enzyme E1
HVO_0651	*pfdB*	CP	prefoldin beta subunit
HVO_0689	*smc*	CP	chromosome segregation protein Smc
HVO_0717	*ftsZ1*	CP	cell division protein FtsZ, type I
HVO_0783	*lon*	CP	ATP-dependent protease La
HVO_0850	*panA*	CP	proteasome-activating nucleotidase
HVO_1091	*psmA1*	CP	proteasome alpha subunit
HVO_1562	*psmB*	CP	proteasome beta subunit
HVO_1957	*panB*	CP	proteasome-activating nucleotidase
HVO_2175	*sph3*	CP	SMC-like protein Sph3
HVO_2619	*samp1, moaD1*	CP	ubiquitin-like modifier protein SAMP1
HVO_2923	*psmA2*	CP	proteasome alpha subunit
HVO_A0180	*sph1*	CP	SMC-like protein Sph1
HVO_B0118	*sph2*	CP	SMC-like protein Sph2
HVO_B0173	*sph4*	CP	SMC-like protein Sph4
HVO_0002	*sec11b*	SEC	signal peptidase I
HVO_0120	*ftsY*	SEC	signal recognition particle receptor FtsY
HVO_0123	*srp54*	SEC	signal recognition particle 54K protein
HVO_0185	*tatCt*	SEC	Sec-independent protein translocase subunit TatCt
HVO_0186	*tatCo*	SEC	Sec-independent protein translocase subunit TatCo
HVO_0437	*secG, sec61b*	SEC	protein translocase subunit SecG
HVO_0718	*secE, sec61c*	SEC	protein translocase subunit SecE
HVO_1027	*tatAo*	SEC	Sec-independent protein translocase subunit TatAo
HVO_1975	*secF*	SEC	protein-export membrane protein SecF
HVO_1976	*secD*	SEC	protein-export membrane protein SecD
HVO_2541	*secY1, sec61a1*	SEC	protein translocase subunit SecY
HVO_A0174	*secY2, sec61a2*	SEC	protein translocase subunit SecY
HVO_0020	*thiQ*	TP	ABC-type transport system ATP-binding protein (probable substrate thiamine)
HVO_0022	*thiB*	TP	ABC-type transport system periplasmic substrate-binding protein (probable substrate thiamine)
HVO_0058	*dppF1*	TP	ABC-type transport system ATP-binding protein (probable substrate dipeptide/oligopeptide)
HVO_0059	*dppD1*	TP	ABC-type transport system ATP-binding protein (probable substrate dipeptide/oligopeptide)
HVO_0060	*dppC1*	TP	ABC-type transport system permease protein (probable substrate dipeptide/oligopeptide)
HVO_0061	*dppB1*	TP	ABC-type transport system permease protein (probable substrate dipeptide/oligopeptide)
HVO_0062	*dppA1*	TP	ABC-type transport system periplasmic substrate-binding protein (probable substrate dipeptide/oligopeptide)
HVO_0084	*amt1*	TP	transport protein (probable substrate ammonium)
HVO_0086	*amt2*	TP	transport protein (probable substrate ammonium)

HVO_0244		TP	transport protein (probable substrate zinc/cadmium)
HVO_0248		TP	transport protein (probable substrate phosphate/sulfate)
HVO_0335	*uraA3*	TP	xanthine/uracil permease family transport protein
HVO_0424		TP	ABC-type transport system ATP-binding protein
HVO_0445	*phnE1*	TP	ABC-type transport system permease protein (probable substrate phosphate/phosphonate)
HVO_0446	*phnC1*	TP	ABC-type transport system ATP-binding protein (probable substrate phosphate/phosphonate)
HVO_0447	*phnD1*	TP	ABC-type transport system periplasmic substrate-binding protein (probable substrate phosphate/phosphonate)
HVO_0506		TP	ABC-type transport system ATP-binding/permease protein
HVO_0530	*tsgA1*	TP	ABC-type transport system periplasmic substrate-binding protein (probable substrate sugar)
HVO_0531	*tsgB1*	TP	ABC-type transport system permease protein (probable substrate sugar)
HVO_0532	*tsgC1*	TP	ABC-type transport system permease protein (probable substrate sugar)
HVO_0534	*tsgD1*	TP	ABC-type transport system ATP-binding protein (probable substrate sugar)
HVO_0544		TP	major facilitator superfamily transporter
HVO_0564	*malE*	TP	ABC-type transport system periplasmic substrate-binding protein (probable substrate maltose)
HVO_0565	*malK*	TP	ABC-type transport system ATP-binding protein (probable substrate maltose)
HVO_0627	*dppDF2*	TP	ABC-type transport system ATP-binding protein (probable substrate dipeptide/oligopeptide)
HVO_0628	*dppA2*	TP	ABC-type transport system periplasmic substrate-binding protein (probable substrate dipeptide/oligopeptide)
HVO_0629	*dppB2*	TP	ABC-type transport system permease protein (probable substrate dipeptide/oligopeptide)
HVO_0630	*dppC2*	TP	ABC-type transport system permease protein (probable substrate dipeptide/oligopeptide)
HVO_0715		TP	sodium/calcium antiporter
HVO_0831	*argK*	TP	ArgK-type transport ATPase
HVO_0891	*nosF*	TP	ABC-type transport system ATP-binding protein (probable substrate copper)
HVO_0892	*nosD*	TP	ABC-type transport system periplasmic substrate-binding protein (probable substrate copper)
HVO_0899	*livJ1*	TP	ABC-type transport system periplasmic substrate-binding protein (probable substrate branched-chain amino acids)
HVO_0902	*livG1*	TP	ABC-type transport system ATP-binding protein (probable substrate branched-chain amino acids)
HVO_0903	*livF1*	TP	ABC-type transport system ATP-binding protein (probable substrate branched-chain amino acids)
HVO_0933		TP	P-type transport ATPase (probable substrate copper/metal cation)
HVO_0940	*cpx*	TP	P-type transport ATPase (probable substrate copper/metal cation)
HVO_1006		TP	ABC-type transport system ATP-binding protein
HVO_1057	*trkH2*	TP	Trk potassium uptake system protein TrkH
HVO_1065	*phaE*	TP	Mrp-type sodium/proton antiporter system subunit E
HVO_1066	*phaD1*	TP	Mrp-type sodium/proton antiporter system subunit D1
HVO_1069	*phaA*	TP	Mrp-type sodium/proton antiporter system subunit A
HVO_1110	*btuF*	TP	ABC-type transport system periplasmic substrate-binding protein (probable substrate cobalamin)
HVO_1112	*btuD*	TP	ABC-type transport system ATP-binding protein (probable substrate cobalamin)
HVO_1165	*mscS1*	TP	mechanosensitive channel protein MscS
HVO_1195	*livG2*	TP	ABC-type transport system ATP-binding protein (probable substrate branched-chain amino acids)
HVO_1196	*livF2*	TP	ABC-type transport system ATP-binding protein (probable substrate branched-chain amino acids)
HVO_1262	*nhaC2*	TP	probable NhaC-type sodium/proton antiporter
HVO_1394	*nhaC3*	TP	probable NhaC-type sodium/proton antiporter
HVO_1398	*tsgC11*	TP	ABC-type transport system permease protein (probable substrate sugar)
HVO_1399	*tsgB11*	TP	ABC-type transport system permease protein (probable substrate sugar)
HVO_1400	*tsgD11*	TP	ABC-type transport system ATP-binding protein (probable substrate sugar)
HVO_1401	*tsgA11*	TP	ABC-type transport system periplasmic substrate-binding protein (probable substrate sugar)
HVO_1441		TP	ABC-type transport system permease protein
HVO_1442		TP	ABC-type transport system permease protein
HVO_1443		TP	ABC-type transport system ATP-binding protein
HVO_1464		TP	ABC-type transport system periplasmic substrate-binding protein (probable substrate iron-III)
HVO_1495	*ptfB*	TP	phosphotransferase system component IIB, fructose-specific
HVO_1499	*ptfC*	TP	phosphotransferase system component IIC, fructose-specific
HVO_1601	*ssuB*	TP	ABC-type transport system ATP-binding protein (probable substrate sulfonate)
HVO_1602	*ssuC*	TP	ABC-type transport system permease protein (probable substrate sulfonate)
HVO_1603	*ssuA*	TP	ABC-type transport system periplasmic substrate-binding protein (probable substrate sulfonate)
HVO_1605	*nhaC5*	TP	probable NhaC-type sodium/proton antiporter
HVO_1659	*mscS2*	TP	mechanosensitive channel protein MscS
HVO_1675	*corA*	TP	magnesium transport protein CorA
HVO_1696		TP	L-lactate permease
HVO_1705		TP	ABC-type transport system periplasmic substrate-binding protein (probable substrate iron-III)
HVO_1707		TP	ABC-type transport system ATP-binding protein (probable substrate iron-III)
HVO_1751	*copA*	TP	P-type transport ATPase (probable substrate copper/metal cation)
HVO_1759		TP	ABC-type transport system permease protein (probable substrate iron-III)
HVO_1760		TP	ABC-type transport system ATP-binding protein (probable substrate iron-III)
HVO_1886		TP	ABC-type transport system ATP-binding protein (probable substrate sulfate/tungstate)
HVO_1888		TP	ABC-type transport system periplasmic substrate-binding protein (probable substrate sulfate/tungstate)
HVO_1916	*kef1*	TP	Kef-type transport system (probable substrate potassium)
HVO_1920	*trnS*	TP	transport protein (probable substrate dicarboxylate)
HVO_1985		TP	ABC-type transport system ATP-binding protein
HVO_1991		TP	ABC-type transport system periplasmic substrate-binding protein (probable substrate iron-III)
HVO_2031	*tsgA12*	TP	ABC-type transport system periplasmic substrate-binding protein (probable substrate sugar)
HVO_2032	*tsgD12*	TP	ABC-type transport system ATP-binding protein (probable substrate sugar)

HVO_2055		TP	probable transport protein (probable polysaccharide biosynthesis transport protein)
HVO_2069		TP	RND superfamily permease
HVO_2079		TP	RND superfamily permease
HVO_2083		TP	ABC-type transport system ATP-binding protein (probable substrate macrolides)
HVO_2084		TP	ABC-type transport system permease protein (probable substrate macrolides)
HVO_2094		TP	ABC-type transport system periplasmic substrate-binding protein (probable substrate iron-III)
HVO_2122	*dppF4*	TP	ABC-type transport system ATP-binding protein (probable substrate dipeptide/oligopeptide)
HVO_2123	*dppD4*	TP	ABC-type transport system ATP-binding protein (probable substrate dipeptide/oligopeptide)
HVO_2124	*dppC4*	TP	ABC-type transport system permease protein (probable substrate dipeptide/oligopeptide)
HVO_2125	*dppB4*	TP	ABC-type transport system permease protein (probable substrate dipeptide/oligopeptide)
HVO_2126	*dppA4*	TP	ABC-type transport system periplasmic substrate-binding protein (probable substrate dipeptide/oligopeptide)
HVO_2163	*lolD*	TP	ABC-type transport system ATP-binding protein (homolog to LolDCE lipoprotein release factor)
HVO_2164	*lolC*	TP	ABC-type transport system permease protein (homolog to LolDCE lipoprotein release factor)
HVO_2165	*lolE*	TP	ABC-type transport system permease protein (homolog to LolDCE lipoprotein release factor)
HVO_2190		TP	gluconate permease
HVO_2249	*uraA2*	TP	xanthine/uracil permease family transport protein
HVO_2324		TP	SSSF family transport protein
HVO_2375	*pstS1*	TP	ABC-type transport system periplasmic substrate-binding protein (probable substrate phosphate)
HVO_2376	*pstC1*	TP	ABC-type transport system permease protein (probable substrate phosphate)
HVO_2377	*pstA1*	TP	ABC-type transport system permease protein (probable substrate phosphate)
HVO_2378	*pstB1*	TP	ABC-type transport system ATP-binding protein (probable substrate phosphate)
HVO_2397	*znuA1*	TP	ABC-type transport system periplasmic substrate-binding protein (probable substrate zinc)
HVO_2398	*znuC1*	TP	ABC-type transport system ATP-binding protein (probable substrate zinc)
HVO_2430	*glnQ*	TP	ABC-type transport system ATP-binding protein (probable substrate glutamine)
HVO_2431	*glnP*	TP	ABC-type transport system permease protein (probable substrate glutamine)
HVO_2432	*glnH*	TP	ABC-type transport system periplasmic substrate-binding protein (probable substrate glutamine)
HVO_2443	*dppD5*	TP	ABC-type transport system ATP-binding protein (probable substrate dipeptide/oligopeptide)
HVO_2444	*dppF5*	TP	ABC-type transport system ATP-binding protein (probable substrate dipeptide/oligopeptide) (nonfunctional)
HVO_2469		TP	transport protein (homolog to dopamine transporter)
HVO_2470		TP	transport protein (homolog to dopamine transporter)
HVO_2500	*cat2*	TP	transport protein (probable substrate cationic amino acids)
HVO_2602		TP	transport protein (probable substrate zinc/cadmium)
HVO_2685	*mscS3*	TP	mechanosensitive channel protein MscS
HVO_2692	*tsgD3*	TP	ABC-type transport system ATP-binding protein (probable substrate sugar)
HVO_2695	*tsgA3*	TP	ABC-type transport system periplasmic substrate-binding protein (probable substrate sugar)
HVO_2745	*mscS5*	TP	mechanosensitive channel protein MscS
HVO_2798	*livJ3*	TP	ABC-type transport system periplasmic substrate-binding protein (probable substrate branched-chain amino acids)
HVO_2800	*livF3*	TP	ABC-type transport system ATP-binding protein (probable substrate branched-chain amino acids)
HVO_2801	*livG3*	TP	ABC-type transport system ATP-binding protein (probable substrate branched-chain amino acids)
HVO_2802	*livM3*	TP	ABC-type transport system permease protein (probable substrate branched-chain amino acids)
HVO_2803	*livH3*	TP	ABC-type transport system permease protein (probable substrate branched-chain amino acids)
HVO_3001		TP	ABC-type transport system permease protein
HVO_3002		TP	ABC-type transport system ATP-binding protein
HVO_A0145	*tsgA4*	TP	ABC-type transport system ATP-binding protein (probable substrate sugar)
HVO_A0154		TP	SSSF family transport protein
HVO_A0283	*tsgA5*	TP	ABC-type transport system periplasmic substrate-binding protein (probable substrate sugar)
HVO_A0293	*potA1*	TP	ABC-type transport system ATP-binding protein (probable substrate spermidine/putrescine)
HVO_A0294	*potA2*	TP	ABC-type transport system ATP-binding protein (probable substrate spermidine/putrescine)
HVO_A0299	*potD*	TP	ABC-type transport system periplasmic substrate-binding protein (probable substrate spermidine/putrescine)
HVO_A0300	*potB*	TP	ABC-type transport system permease protein (probable substrate spermidine/putrescine)
HVO_A0323		TP	ABC-type transport system ATP-binding protein
HVO_A0336	*dppDF7*	TP	ABC-type transport system ATP-binding protein (probable substrate dipeptide/oligopeptide)
HVO_A0339	*dppA7*	TP	ABC-type transport system periplasmic substrate-binding protein (probable substrate dipeptide/oligopeptide)
HVO_A0380	*dppA8*	TP	ABC-type transport system periplasmic substrate-binding protein (probable substrate dipeptide/oligopeptide)
HVO_A0383	*dppD8*	TP	ABC-type transport system ATP-binding protein (probable substrate dipeptide/oligopeptide)
HVO_A0425	*dppF9*	TP	ABC-type transport system ATP-binding protein (probable substrate dipeptide/oligopeptide)
HVO_A0426	*dppD9*	TP	ABC-type transport system ATP-binding protein (probable substrate dipeptide/oligopeptide)
HVO_A0428	*dppA9*	TP	ABC-type transport system periplasmic substrate-binding protein (probable substrate dipeptide/oligopeptide)
HVO_A0451	*arsB4*	TP	transport protein (probable substrate arsenite/antimonite)
HVO_A0477	*pstS2*	TP	ABC-type transport system periplasmic substrate-binding protein (probable substrate phosphate)
HVO_A0491	*tsgD6*	TP	ABC-type transport system ATP-binding protein (probable substrate sugar)
HVO_A0493	*tsgB6*	TP	ABC-type transport system permease protein (probable substrate sugar)
HVO_A0494	*tsgA6*	TP	ABC-type transport system periplasmic substrate-binding protein (probable substrate sugar)
HVO_A0541		TP	ABC-type transport system periplasmic substrate-binding protein (probable substrate iron-III)
HVO_A0557		TP	ABC-type transport system periplasmic substrate-binding protein (probable substrate iron-III)
HVO_A0576	*livJ5*	TP	ABC-type transport system periplasmic substrate-binding protein (probable substrate branched-chain amino acids)
HVO_A0580	*livF5*	TP	ABC-type transport system ATP-binding protein (probable substrate branched-chain amino acids)
HVO_A0606		TP	major facilitator superfamily transporter
HVO_A0611	*znuA2*	TP	ABC-type transport system periplasmic substrate-binding protein (probable substrate zinc)
HVO_A0624	*cadA, zntA1*	TP	P-type transport ATPase (probable substrate zinc/cadmium)

HVO_B0021	*dppA10*	TP	ABC-type transport system periplasmic substrate-binding protein (probable substrate dipeptide/oligopeptide)
HVO_B0024	*dppDF10*	TP	ABC-type transport system ATP-binding protein (probable substrate dipeptide/oligopeptide)
HVO_B0037	*tsgE7*	TP	ABC-type transport system ATP-binding protein (probable substrate sugar)
HVO_B0038	*tsgD7*	TP	ABC-type transport system ATP-binding protein (probable substrate sugar)
HVO_B0047		TP	ABC-type transport system periplasmic substrate-binding protein (probable substrate iron-III)
HVO_B0067	*cat7*	TP	transport protein (probable substrate cationic amino acids)
HVO_B0078	*dppF11*	TP	ABC-type transport system ATP-binding protein (probable substrate dipeptide/oligopeptide)
HVO_B0082	*dppA11*	TP	ABC-type transport system periplasmic substrate-binding protein (probable substrate dipeptide/oligopeptide)
HVO_B0089	*dppF12*	TP	ABC-type transport system ATP-binding protein (probable substrate dipeptide/oligopeptide)
HVO_B0093	*dppA12*	TP	ABC-type transport system periplasmic substrate-binding protein (probable substrate dipeptide/oligopeptide)
HVO_B0106	*tsgA8*	TP	ABC-type transport system periplasmic substrate-binding protein (probable substrate sugar)
HVO_B0124	*dppC13*	TP	ABC-type transport system permease protein (probable substrate dipeptide/oligopeptide)
HVO_B0134		TP	ABC-type transport system ATP-binding protein
HVO_B0144		TP	ABC-type transport system periplasmic substrate-binding protein (probable substrate iron-III)
HVO_B0177	*cbiO3*	TP	ABC-type transport system ATP-binding protein (probable substrate cobalt)
HVO_B0184	*dppA14*	TP	ABC-type transport system periplasmic substrate-binding protein (probable substrate dipeptide/oligopeptide)
HVO_B0197		TP	ABC-type transport system permease protein (probable substrate iron-III)
HVO_B0198		TP	ABC-type transport system periplasmic substrate-binding protein (probable substrate iron-III)
HVO_B0217	*livJ6*	TP	ABC-type transport system periplasmic substrate-binding protein (probable substrate branched-chain amino acids)
HVO_B0227	*tsgD9*	TP	ABC-type transport system ATP-binding protein (probable substrate sugar)
HVO_B0228	*tsgA9*	TP	ABC-type transport system periplasmic substrate-binding protein (probable substrate sugar)
HVO_B0276		TP	DMT superfamily transport protein
HVO_B0292	*ugpB*	TP	ABC-type transport system periplasmic substrate-binding protein (probable substrate glycerol-3-phosphate)
HVO_B0295	*ugpC*	TP	ABC-type transport system ATP-binding protein (probable substrate glycerol-3-phosphate)
HVO_B0316	*tsgD13*	TP	ABC-type transport system ATP-binding protein (substrate glucose)
HVO_B0318	*tsgA13*	TP	ABC-type transport system periplasmic substrate-binding protein (substrate glucose)
HVO_B0328	*dppA15*	TP	ABC-type transport system periplasmic substrate-binding protein (probable substrate dipeptide/oligopeptide)
HVO_B0335		TP	major facilitator superfamily transporter
HVO_B0369		TP	ABC-type transport system periplasmic substrate-binding protein (probable substrate molybdate)
HVO_C0072	*dppC16*	TP	ABC-type transport system permease protein (probable substrate dipeptide/oligopeptide)
HVO_C0073	*dppB16*	TP	ABC-type transport system permease protein (probable substrate dipeptide/oligopeptide)
HVO_C0074	*dppDF16*	TP	ABC-type transport system ATP-binding protein (probable substrate dipeptide/oligopeptide)
HVO_C0075	*dppA16*	TP	ABC-type transport system periplasmic substrate-binding protein (probable substrate dipeptide/oligopeptide)
Unassigned function			
HVO_0017		CHY	conserved hypothetical protein
HVO_0056		CHY	conserved hypothetical protein
HVO_0095		CHY	conserved hypothetical protein
HVO_0103		CHY	conserved hypothetical protein
HVO_0127		CHY	conserved hypothetical protein
HVO_0140		CHY	conserved hypothetical protein
HVO_0154		CHY	conserved hypothetical protein
HVO_0192		CHY	conserved hypothetical protein
HVO_0196		CHY	conserved hypothetical protein
HVO_0229		CHY	conserved hypothetical protein
HVO_0234		CHY	conserved hypothetical protein
HVO_0306		CHY	conserved hypothetical protein
HVO_0307		CHY	conserved hypothetical protein
HVO_0336		CHY	conserved hypothetical protein (nonfunctional)
HVO_0382		CHY	conserved hypothetical protein
HVO_0399		CHY	conserved hypothetical protein
HVO_0400		CHY	conserved hypothetical protein
HVO_0407		CHY	conserved hypothetical protein
HVO_0413		CHY	conserved hypothetical protein
HVO_0423		CHY	conserved hypothetical protein
HVO_0471		CHY	conserved hypothetical protein
HVO_0475		CHY	conserved hypothetical protein
HVO_0476		CHY	conserved hypothetical protein
HVO_0500		CHY	conserved hypothetical protein
HVO_0508		CHY	conserved hypothetical protein
HVO_0539		CHY	conserved hypothetical protein
HVO_0545		CHY	conserved hypothetical protein
HVO_0547		CHY	conserved hypothetical protein
HVO_0583		CHY	conserved hypothetical protein
HVO_0656		CHY	conserved hypothetical protein
HVO_0703		CHY	conserved hypothetical protein
HVO_0711		CHY	conserved hypothetical protein
HVO_0734		CHY	conserved hypothetical protein
HVO_0738		CHY	conserved hypothetical protein
HVO_0739		CHY	conserved hypothetical protein
HVO_0753		CHY	conserved hypothetical protein

HVO_0764		CHY	conserved hypothetical protein
HVO_0797		CHY	conserved hypothetical protein
HVO_0805		CHY	conserved hypothetical protein
HVO_0838		CHY	conserved hypothetical protein
HVO_0843		CHY	conserved hypothetical protein
HVO_0845		CHY	conserved hypothetical protein
HVO_0880		CHY	conserved hypothetical protein
HVO_0908		CHY	conserved hypothetical protein
HVO_0918		CHY	conserved hypothetical protein
HVO_0939		CHY	conserved hypothetical protein
HVO_0941		CHY	conserved hypothetical protein
HVO_0953		CHY	conserved hypothetical protein
HVO_0954		CHY	conserved hypothetical protein
HVO_0958		CHY	conserved hypothetical protein
HVO_0959		CHY	conserved hypothetical protein
HVO_0989		CHY	conserved hypothetical protein
HVO_0995		CHY	conserved hypothetical protein
HVO_1023		CHY	conserved hypothetical protein
HVO_1059		CHY	conserved hypothetical protein
HVO_1080		CHY	conserved hypothetical protein
HVO_1095		CHY	conserved hypothetical protein
HVO_1102		CHY	conserved hypothetical protein
HVO_1103		CHY	conserved hypothetical protein
HVO_1129		CHY	conserved hypothetical protein
HVO_1130		CHY	conserved hypothetical protein
HVO_1133		CHY	conserved hypothetical protein
HVO_1134		CHY	conserved hypothetical protein
HVO_1135		CHY	conserved hypothetical protein
HVO_1156		CHY	conserved hypothetical protein
HVO_1176		CHY	conserved hypothetical protein
HVO_1187		CHY	conserved hypothetical protein
HVO_1202		CHY	conserved hypothetical protein
HVO_1208		CHY	conserved hypothetical protein
HVO_1240		CHY	conserved hypothetical protein
HVO_1242		CHY	conserved hypothetical protein
HVO_1249		CHY	conserved hypothetical protein
HVO_1256		CHY	conserved hypothetical protein
HVO_1258		CHY	conserved hypothetical protein
HVO_1259		CHY	conserved hypothetical protein
HVO_1261		CHY	conserved hypothetical protein
HVO_1263		CHY	conserved hypothetical protein
HVO_1264		CHY	conserved hypothetical protein
HVO_1268		CHY	conserved hypothetical protein
HVO_1274		CHY	conserved hypothetical protein
HVO_1293		CHY	conserved hypothetical protein
HVO_1301		CHY	conserved hypothetical protein
HVO_1332		CHY	conserved hypothetical protein
HVO_1388		CHY	conserved hypothetical protein
HVO_1424		CHY	conserved hypothetical protein
HVO_1425		CHY	conserved hypothetical protein
HVO_1437		CHY	conserved hypothetical protein
HVO_1457		CHY	conserved hypothetical protein
HVO_1485		CHY	conserved hypothetical protein
HVO_1492		CHY	conserved hypothetical protein
HVO_1513		CHY	conserved hypothetical protein
HVO_1542		CHY	conserved hypothetical protein
HVO_1548		CHY	conserved hypothetical protein
HVO_1563		CHY	conserved hypothetical protein
HVO_1564		CHY	conserved hypothetical protein
HVO_1567		CHY	conserved hypothetical protein
HVO_1577		CHY	conserved hypothetical protein
HVO_1580		CHY	conserved hypothetical protein
HVO_1591		CHY	conserved hypothetical protein
HVO_1595		CHY	conserved hypothetical protein
HVO_1597		CHY	conserved hypothetical protein
HVO_1609		CHY	conserved hypothetical protein
HVO_1610		CHY	conserved hypothetical protein
HVO_1615		CHY	conserved hypothetical protein
HVO_1623		CHY	conserved hypothetical protein
HVO_1625		CHY	conserved hypothetical protein

HVO_1629		CHY	conserved hypothetical protein
HVO_1636		CHY	conserved hypothetical protein
HVO_1673		CHY	conserved hypothetical protein
HVO_1685		CHY	conserved hypothetical protein
HVO_1693		CHY	conserved hypothetical protein
HVO_1694		CHY	conserved hypothetical protein
HVO_1735		CHY	conserved hypothetical protein
HVO_1740		CHY	conserved hypothetical protein
HVO_1741		CHY	conserved hypothetical protein
HVO_1745		CHY	conserved hypothetical protein
HVO_1749		CHY	conserved hypothetical protein
HVO_1783		CHY	conserved hypothetical protein
HVO_1806		CHY	conserved hypothetical protein
HVO_1808		CHY	conserved hypothetical protein
HVO_1812		CHY	conserved hypothetical protein
HVO_1819		CHY	conserved hypothetical protein
HVO_1824		CHY	conserved hypothetical protein
HVO_1825		CHY	conserved hypothetical protein
HVO_1868		CHY	conserved hypothetical protein
HVO_1880		CHY	conserved hypothetical protein
HVO_1894		CHY	conserved hypothetical protein
HVO_1944		CHY	conserved hypothetical protein
HVO_1945		CHY	conserved hypothetical protein
HVO_1949		CHY	conserved hypothetical protein
HVO_1973		CHY	conserved hypothetical protein
HVO_1988		CHY	conserved hypothetical protein
HVO_1995		CHY	conserved hypothetical protein
HVO_2006		CHY	conserved hypothetical protein
HVO_2015		CHY	conserved hypothetical protein
HVO_2016		CHY	conserved hypothetical protein
HVO_2017		CHY	conserved hypothetical protein
HVO_2018		CHY	conserved hypothetical protein
HVO_2022		CHY	conserved hypothetical protein
HVO_2024		CHY	conserved hypothetical protein
HVO_2047		CHY	conserved hypothetical protein
HVO_2066		CHY	conserved hypothetical protein
HVO_2070		CHY	conserved hypothetical protein
HVO_2071		CHY	conserved hypothetical protein
HVO_2074		CHY	conserved hypothetical protein
HVO_2076		CHY	conserved hypothetical protein
HVO_2080		CHY	conserved hypothetical protein
HVO_2082		CHY	conserved hypothetical protein
HVO_2120		CHY	conserved hypothetical protein
HVO_2135		CHY	conserved hypothetical protein
HVO_2160		CHY	conserved hypothetical protein
HVO_2161		CHY	conserved hypothetical protein
HVO_2167		CHY	conserved hypothetical protein
HVO_2169		CHY	conserved hypothetical protein
HVO_2172		CHY	conserved hypothetical protein
HVO_2203		CHY	conserved hypothetical protein
HVO_2241		CHY	conserved hypothetical protein
HVO_2248		CHY	conserved hypothetical protein
HVO_2255		CHY	conserved hypothetical protein
HVO_2263		CHY	conserved hypothetical protein
HVO_2272		CHY	conserved hypothetical protein
HVO_2276		CHY	conserved hypothetical protein
HVO_2286		CHY	conserved hypothetical protein
HVO_2291_A		CHY	conserved hypothetical protein
HVO_2325		CHY	conserved hypothetical protein
HVO_2333		CHY	conserved hypothetical protein
HVO_2334		CHY	conserved hypothetical protein
HVO_2347		CHY	conserved hypothetical protein
HVO_2369		CHY	conserved hypothetical protein
HVO_2417		CHY	conserved hypothetical protein
HVO_2418		CHY	conserved hypothetical protein
HVO_2513		CHY	conserved hypothetical protein
HVO_2519		CHY	conserved hypothetical protein
HVO_2533		CHY	conserved hypothetical protein
HVO_2537		CHY	conserved hypothetical protein
HVO_2565		CHY	conserved hypothetical protein

HVO_2630		CHY	conserved hypothetical protein
HVO_2635		CHY	conserved hypothetical protein
HVO_2637		CHY	conserved hypothetical protein
HVO_2640		CHY	conserved hypothetical protein
HVO_2689		CHY	conserved hypothetical protein
HVO_2770		CHY	conserved hypothetical protein
HVO_2793		CHY	conserved hypothetical protein
HVO_2796		CHY	conserved hypothetical protein
HVO_2832		CHY	conserved hypothetical protein (nonfunctional)
HVO_2847		CHY	conserved hypothetical protein
HVO_2860		CHY	conserved hypothetical protein
HVO_2861		CHY	conserved hypothetical protein
HVO_2872		CHY	conserved hypothetical protein
HVO_2883		CHY	conserved hypothetical protein
HVO_2884		CHY	conserved hypothetical protein
HVO_2914		CHY	conserved hypothetical protein
HVO_2921		CHY	conserved hypothetical protein
HVO_2972		CHY	conserved hypothetical protein
HVO_A0002		CHY	conserved hypothetical protein
HVO_A0010		CHY	conserved hypothetical protein
HVO_A0021		CHY	conserved hypothetical protein
HVO_A0031		CHY	conserved hypothetical protein
HVO_A0034		CHY	conserved hypothetical protein
HVO_A0085		CHY	conserved hypothetical protein
HVO_A0133		CHY	conserved hypothetical protein
HVO_A0164		CHY	conserved hypothetical protein
HVO_A0181		CHY	conserved hypothetical protein
HVO_A0242		CHY	conserved hypothetical protein
HVO_A0256		CHY	conserved hypothetical protein
HVO_A0324		CHY	conserved hypothetical protein
HVO_A0350		CHY	conserved hypothetical protein
HVO_A0356		CHY	conserved hypothetical protein (nonfunctional)
HVO_A0400		CHY	conserved hypothetical protein
HVO_A0406		CHY	conserved hypothetical protein
HVO_A0418		CHY	conserved hypothetical protein
HVO_A0432		CHY	conserved hypothetical protein
HVO_A0453		CHY	conserved hypothetical protein
HVO_A0466		CHY	conserved hypothetical protein
HVO_A0476		CHY	conserved hypothetical protein
HVO_A0498		CHY	conserved hypothetical protein
HVO_A0499		CHY	conserved hypothetical protein
HVO_A0619		CHY	conserved hypothetical protein
HVO_A0627		CHY	conserved hypothetical protein
HVO_A0631		CHY	conserved hypothetical protein
HVO_B0006		CHY	conserved hypothetical protein
HVO_B0053		CHY	conserved hypothetical protein
HVO_B0055		CHY	conserved hypothetical protein
HVO_B0064		CHY	conserved hypothetical protein
HVO_B0084		CHY	conserved hypothetical protein
HVO_B0117		CHY	conserved hypothetical protein
HVO_B0131		CHY	conserved hypothetical protein
HVO_B0135		CHY	conserved hypothetical protein
HVO_B0153_A		CHY	conserved hypothetical protein
HVO_B0156		CHY	conserved hypothetical protein
HVO_B0171		CHY	conserved hypothetical protein
HVO_B0174		CHY	conserved hypothetical protein
HVO_B0194		CHY	conserved hypothetical protein
HVO_B0215		CHY	conserved hypothetical protein
HVO_B0223		CHY	conserved hypothetical protein
HVO_B0324		CHY	conserved hypothetical protein
HVO_B0354		CHY	conserved hypothetical protein
HVO_C0035		CHY	homolog to transfer complex protein
HVO_C0036		CHY	conserved hypothetical protein
HVO_C0041		CHY	conserved hypothetical protein
HVO_C0043		CHY	conserved hypothetical protein
HVO_C0046		CHY	conserved hypothetical protein
HVO_C0050		CHY	conserved hypothetical protein
HVO_C0081		CHY	conserved hypothetical protein
HVO_0295		HY	hypothetical protein
HVO_0951		HY	hypothetical protein

HVO_1981		HY	hypothetical protein
HVO_2266		HY	hypothetical protein
HVO_2267		HY	hypothetical protein
HVO_A0012		HY	hypothetical protein
HVO_A0022		HY	hypothetical protein
HVO_A0039		HY	hypothetical protein
HVO_B0270		HY	hypothetical protein
HVO_C0054		HY	hypothetical protein
