# Supplementary material for: Data in support of global role of the membrane protease LonB in Archaea: Potential protease targets revealed by quantitative proteome analysis of a lonB mutant in Haloferax volcanii
Source: Data Brief. 2015 May 7;4:50–3. doi: 10.1016/j.dib.2015.04.013 (PMC4510384; doi:10.1016/j.dib.2015.04.013)
Supplement: Supplementary file 1 — Supplementary material: Supplementary Table S3(1–4): Proteome Discoverer database search of all the replicates of wt, HVLON3 and HVABI strains. The data in the different files is distributed as follow: S3-1. Cytoplasmic fraction exponential phase. S3-2. Cytoplasmic fraction stationary phase. S3-3. Membrane fraction exponential phase. S3-4. Membrane fraction stationary phase. The replicates for each condition are named A to D in the file sheet. Accession: unique identifier assigned to the protein by the H. volcanii FASTA database. Description: name of the protein. ∑Coverage: percentage of the protein sequence covered by identified peptides. ∑# Proteins: number of identified proteins in the protein group of a master protein. ∑# Unique Peptides: number of peptide sequences unique to a protein group. ∑# Peptides: total number of peptide matches found during the search. ∑# PSMs: total number of identified peptide sequences (peptide spectrum matches) for the protein, including those redundantly identified. Area: average area of the three unique peptides with the largest peak area. Score: protein score, which is the sum of the scores of the individual peptides. A2: Results from Sequest HT. A4: Results from MSAmanda. # AAs: sequence length of the protein. MW [Da]: calculated molecular weight of the protein. calc. pI: theoretically calculated isoelectric point. [file mmc1.zip › Table S2.pdf]

Table S2. List of proteins detected as unique in the wt and HVLON3 strains in Exp and St phase.

Proteins only in "*wt* Exp"	Proteins only in "Hvlon3 Exp"	Proteins only in "*wt* St"	Proteins only in "Hvlon3 St"
HVO_0110	HVO_0076	HVO_0009	HVO_0084
HVO_0255	HVO_0099	HVO_0038	HVO_0086
HVO_0279	HVO_0275	HVO_0066	HVO_0127
HVO_0301	HVO_0651	HVO_0095	HVO_0153
HVO_0309	HVO_0935	HVO_0143	HVO_0213
HVO_0405	HVO_0969	HVO_0150	HVO_0295
HVO_0539	HVO_0977	HVO_0173	HVO_0336
HVO_0765	HVO_1013	HVO_0207	HVO_0351
HVO_0781	HVO_1138	HVO_0286	HVO_0364
HVO_1195	HVO_1196	HVO_0303	HVO_0382
HVO_1296	HVO_1223	HVO_0318	HVO_0413
HVO_1462	HVO_1263	HVO_0390	HVO_0419
HVO_1492	HVO_1283	HVO_0409	HVO_0459
HVO_1584	HVO_1382	HVO_0448	HVO_0588
HVO_1622	HVO_1463	HVO_0450	HVO_0633
HVO_1694	HVO_1483	HVO_0483	HVO_0736
HVO_1718	HVO_1839	HVO_0486	HVO_0748
HVO_1745	HVO_1859	HVO_0547	HVO_0805
HVO_1825	HVO_1953	HVO_0567	HVO_0939
HVO_1838	HVO_1999	HVO_0611	HVO_0941
HVO_2076	HVO_2022	HVO_0634	HVO_0952
HVO_2198	HVO_2073	HVO_0656	HVO_1007
HVO_2360	HVO_2096	HVO_0682	HVO_1173
HVO_2565	HVO_2135	HVO_0696	HVO_1259
HVO_2628	HVO_2141	HVO_0734	HVO_1333
HVO_2683	HVO_2147	HVO_0740	HVO_1377
HVO_2747	HVO_2291_A	HVO_0755	HVO_1388
HVO_2859	HVO_2307	HVO_0771	HVO_1441
HVO_2876	HVO_2349	HVO_0814	HVO_1580
HVO_2905	HVO_2444	HVO_0902	HVO_1713
HVO_3005	HVO_2502	HVO_0903	HVO_1752
HVO_3008	HVO_2655	HVO_0918	HVO_1759
HVO_A0045	HVO_2685	HVO_1041	HVO_1822
HVO_A0162	HVO_2699	HVO_1160	HVO_1853
HVO_A0346	HVO_2727	HVO_1202	HVO_1862
HVO_A0401	HVO_2986	HVO_1208	HVO_1908
HVO_A0569	HVO_A0018	HVO_1209	HVO_1911
HVO_B0045	HVO_A0022	HVO_1265	HVO_1926
HVO_B0243	HVO_A0031	HVO_1272	HVO_1929
HVO_B0263	HVO_A0157	HVO_1279	HVO_2012
HVO_B0361	HVO_A0163	HVO_1301	HVO_2088
HVO_B0369	HVO_A0218	HVO_1314	HVO_2090_A
HVO_C0036	HVO_A0238	HVO_1341	HVO_2222
	HVO_A0260	HVO_1636	HVO_2249
	HVO_A0326	HVO_1659	HVO_2281
	HVO_A0453	HVO_1726	HVO_2323
	HVO_A0499	HVO_1740	HVO_2325
	HVO_A0561	HVO_1779	HVO_2334
	HVO_A0580	HVO_1803	HVO_2385
	HVO_A0606	HVO_2062	HVO_2443
	HVO_A0633	HVO_2091	HVO_2461
	HVO_B0076	HVO_2272	HVO_2468
	HVO_B0085	HVO_2275	HVO_2534
	HVO_B0124	HVO_2328	HVO_2571
	HVO_B0174	HVO_2363	HVO_2577
	HVO_B0197	HVO_2370	HVO_2803
	HVO_B0264	HVO_2494	HVO_2827
	HVO_B0267	HVO_2537	HVO_2847
	HVO_B0325	HVO_2681	HVO_2848
	HVO_C0035	HVO_2797	HVO_2849
	HVO_C0053	HVO_2832	HVO_2886
		HVO_2835	HVO_2894
		HVO_2875	HVO_A0034
		HVO_2972	HVO_A0126
		HVO_2977	HVO_A0127
		HVO_2979	HVO_A0293

		HVO_A0009	HVO_A0295
		HVO_A0010	HVO_A0295_A
		HVO_A0082	HVO_A0299
		HVO_A0085	HVO_A0300
		HVO_A0164	HVO_A0323
		HVO_A0209	HVO_A0324
		HVO_A0256	HVO_A0383
		HVO_A0270	HVO_A0418
		HVO_A0277	HVO_A0440
		HVO_A0332	HVO_A0493
		HVO_A0362	HVO_A0517
		HVO_A0400	HVO_A0523
		HVO_A0434	HVO_A0535
		HVO_A0505	HVO_A0584
		HVO_A0521	HVO_A0589
		HVO_A0536	HVO_B0053
		HVO_A0537	HVO_B0067
		HVO_A0545	HVO_B0078
		HVO_A0555	HVO_B0096
		HVO_A0571	HVO_B0153_A
		HVO_A0591	HVO_B0156
		HVO_B0006	HVO_B0171
		HVO_B0027	HVO_B0241
		HVO_B0031	HVO_B0270
		HVO_B0048	HVO_B0276
		HVO_B0069	HVO_B0300
		HVO_B0089	HVO_B0316
		HVO_B0110	HVO_B0319
		HVO_B0119	HVO_B0352
		HVO_B0146	
		HVO_B0179	
		HVO_B0196	
		HVO_B0205	
		HVO_B0216	
		HVO_B0335	
		HVO_B0338	
		HVO_C0073	
